# Hybridogenesis and a potential case of R2 non-LTR retrotransposon horizontal transmission in *Bacillus* stick insects (Insecta Phasmida)

**DOI:** 10.1038/srep41946

**Published:** 2017-02-06

**Authors:** Claudia Scavariello, Andrea Luchetti, Francesco Martoni, Livia Bonandin, Barbara Mantovani

**Affiliations:** 1 Dipartimento di Scienze Biologiche, Geologiche e Ambientali, Università di Bologna, Bologna, Italy; 2 Bio-Protection Research Centre, Lincoln University, Lincoln 7647, New Zealand

## Abstract

Horizontal transfer (HT) is an event in which the genetic material is transferred from one species to another, even if distantly related, and it has been demonstrated as a possible essential part of the lifecycle of transposable elements (TEs). However, previous studies on the non-LTR R2 retrotransposon, a metazoan-wide distributed element, indicated its vertical transmission since the Radiata-Bilateria split. Here we present the first possible instances of R2 HT in stick insects of the genus *Bacillus* (Phasmida). Six R2 elements were characterized in the strictly bisexual subspecies *B. grandii grandii, B. grandii benazzii* and *B. grandii maretimi* and in the obligatory parthenogenetic taxon *B. atticus*. These elements were compared with those previously retrieved in the facultative parthenogenetic species *B. rossius*. Phylogenetic inconsistencies between element and host taxa, and age *versus* divergence analyses agree and support at least two HT events. These HT events can be explained by taking into consideration the complex *Bacillus* reproductive biology, which includes also hybridogenesis, gynogenesis and androgenesis. Through these non-canonical reproductive modes, R2 elements may have been transferred between *Bacillus* genomes. Our data suggest, therefore, a possible role of hybridization for TEs survival and the consequent reshaping of involved genomes.

Transposable elements (TEs) are DNA sequences that are able to self-replicate and move within a genome^1^. TEs can be divided into two classes on the basis of their transposition mechanism. Class I elements, also known as retrotransposons, move by reverse transcription of an RNA intermediate. The presence/absence of long terminal repeats (LTRs) at the element ends further distinguishes LTR and non-LTR retroelements. Non-LTR TEs include long interspersed elements (LINEs) and non-autonomous short interspersed elements (SINEs)^2^. Class II elements, or transposons, move mainly via a mechanism of DNA sequence excision and insertion, although a few of them exploit a rolling-circle mechanism^1^.

The rule of TE vertical inheritance (i.e. the transmission of genetic material from parents to offspring through sexual or asexual reproduction) may be broken by horizontal transfer (HT) events between lineages^3^,^4^. Horizontal transfer is the passage of genetic material between reproductively isolated taxa and it has been proposed as an essential part of the lifecycle of some TE types. TEs are, in fact, usually subject to host’s suppression mechanisms, limiting their mobility and their copy number expansion, and to stochastic losses. In this view, HT events can be considered as an opportunity for TEs to invade a new genome and persist in evolution^5^. HT is apparently more frequent among Class II than Class I TEs. Among the latter, it is more frequent for LTR retrotransposons. This is probably linked to the different transposition mechanisms used by the two classes. DNA transposons have a more stable double-stranded DNA intermediate, while retrotransposons have a relatively unstable RNA intermediate that is reverse-transcribed directly into the chromosomal target site, so that the possible transfer outside the cell appears unlikely. Yet, among Class I elements, there is a small group of LTR retrotransposons (as the *Gypsy* elements) that are similar to retroviruses in terms of replication mechanism and structural organization which may successfully mediate the transfer^6^. Concerning non-LTR retrotransposons, previous analyses assigned these elements to 11 distinct clades but no evidence for HTs was detected within or between these clades during the past 600 Myr^7^,^8^. However, some cases of HT involving both LINEs (CR1, R4, L1 and BovB)^9^,^10^,^11^ and SINEs (Sauria and RUDI)^12^,^13^ have been suggested.

Mechanisms leading to HT are still unclear, although different ways have been observed/suggested: from TEs intrinsic features (such as the presence of retrovirus-like proteins) to vector-mediated transmission and to host-parasite and/or trophic relationship^6^,^12^,^14^,^15^,^16^,^17^. HT has also been attributed to hybridization/introgression events^18^,^19^ linking, therefore, possible TE lateral transfers to specific reproductive biological traits of involved taxa.

Among non-LTR retrotransposons, R2 is probably one of the most investigated element. It has a specific insertion site into the repeated 28S rRNA genes. R2 has a single open reading frame (ORF) flanked by two untranslated sequences (UTRs) of variable length. The translated protein comprises the central reverse transcriptase (RT) domain, the DNA-binding motifs at the N-terminus and the restriction enzyme-like endonuclease (RLE) domain at the C-terminus^20^. Phylogenetic analyses demonstrated that R2 elements can be divided into four main clades (R2-A, -B, -C and -D), in agreement with the number and configuration of N-terminal end zinc-finger motifs, and that the element is present in the animal Kingdom since the Radiata-Bilateria split^21^,^22^,^23^. R2 is subject to significant changes in copy number, even within a single species, due to its rapid turnover^24^,^25^. This may account for the overall phylogenetic incongruence with host species: an R2 phylogenetic analysis across metazoan, in fact, suggested that paralogous lineages replacements and extinctions during the long-term evolution of this non-LTR element may explain its distribution, without assuming horizontal transfer events^22^. A further study suggested that also ancestral libraries of paralogous lineages may account for the observed diversity of multiple lineages within a single genome^23^.

The genus *Bacillus* includes three Mediterranean species of stick insects: *B. rossius* (with eight subspecies), *B. grandii* (with three subspecies) and *B. atticus* (with three karyological/allozyme races). Their taxonomy, systematics and phylogeny are well established and inferred from nuclear (allozyme, centromeric satellite DNA) and mitochondrial markers, as well as from karyological and cytological analyses^26^. The evolutionary history of the genus shows an ancestral divergence, about 22.5 Myr ago, that separated the lineage leading to *B. rossius* from the other ones. Then, around 17 Myr ago, the *B. grandii*/*B. atticus* clade radiated, ending with a paraphyletic relationship between the two species. In fact, the *B. grandii grandii* subspecies appears more related to *B. atticus* than to the *B. grandii benazzii*/*B. grandii maretimi* clade^26^,^27^. *Bacillus* species show a wide range of reproductive strategies, from strict bisexuality (in *B. grandii*) to facultative (*B. rossius*) or obligatory (*B. atticus*) parthenogenesis. Moreover, these species gave origin to inter-specific hybrid taxa, reproducing either through obligate parthenogenesis or hybridogenesis^26^. Hybridogenesis, or hemiclonal reproduction, takes place when the hybrid individual (often a female) discards the paternal haploset during meiosis. The hybrid condition is then restored through fertilization of the egg containing the maternal unrecombined haploset with a paternal sperm^26^. *Bacillus* hybridogenetic strains involve *B. rossius* as maternal lineage and *B. g. grandii* or *B. g. benazzii* as paternal lineage^26^. In these taxa, further, natural androgenesis (i.e. offspring with full paternal nuclear genome derived from two sperms of fertilizing males) was also demonstrated for the first time in the Animal Kingdom^28^. Hybridogenesis and androgenesis are known to occur in both vertebrates and invertebrates where also a peculiar social hybridogenetic system has been demonstrated^29^,^30^,^31^.

We recently analyzed R2 in stick insects of the genus *Bacillus*, addressing the evolutionary dynamics of their insertions in different reproductive strategies^32^,^33^, as well as the coevolution of multiple lineages in the genome of *B. rossius*^34^. In order to have a full picture of R2 evolution in the *Bacillus* species complex, we isolated and characterized R2 from the obligatory parthenogenetic *B. atticus* and the strictly gonochoric *B. grandii*. The analyses of the evolutionary history of all retrieved *Bacillus* R2 lineages suggest the occurrence of HT events that can be explained taking into account the hybridogenetic mechanism.

## Results

### R2 sequences characterization

**Figure 1:**
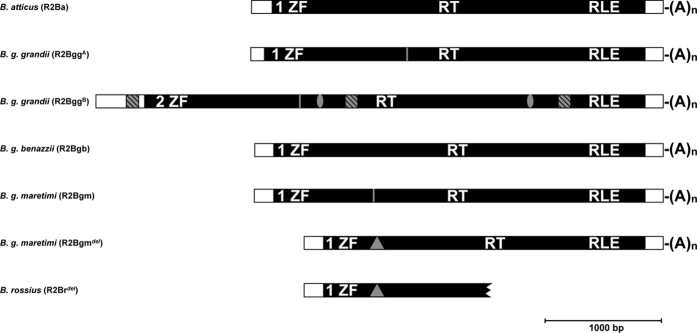
Schematic representation of presently characterized R2 elements in the *Bacillus* genus. For comparison, also the fragment representing R2Br^*del*^ has been reported. Black boxes indicate the open reading frame (ORF) with the zinc finger (ZF), reverse transcriptase (RT) and restriction enzyme-like endonuclease (RLE) domains. Vertical grey lines indicate frameshift mutations; ovals represent stop codon(s); grey squares represent duplications and triangles represent large deletions.

Structures and features of newly obtained R2 elements are given in Fig. 1. One R2 element was found in *B. atticus* (R2Ba); it is 3507 bp long, excluding the 3′ terminal poly-(A) tail of nine nucleotides, with an ORF of 3177 bp encoding a protein of 1058 amino acids. Two R2 elements were isolated from *B. g. grandii*. R2Bgg^A^ is 3513 bp long, excluding the poly-(A) tail of seven nucleotides and shows two overlapping ORFs of 1545 bp and 1698 bp. The analysis of the two proteins suggests they are actually part of a 3243 bp long ORF that encodes a protein of 1079 amino acids in which a frame shift mutation occurred (at pos. 1726). The same can be observed for the second *B. g. grandii* element (R2Bgg^B^), that is 4832 bp long excluding the poly-(A) tail, with a putative ORF of 4264 bp in which a frameshift mutation occurred (at pos. 2191). This element also shows two stop codons (pos. 2913 and pos. 4722) and three sequence duplications of 333 bp, 68 bp and 57 bp, respectively. The first and second duplications are within the ORF while the third one is in the 5′ UTR. The element found in *B. g. benazzii* (R2Bgb) is 3479 bp long, excluding the poly-(A) tail, with an ORF of 3165 bp encoding a protein of 1054 amino acids. In the genome of *B. g. maretimi*, two R2 elements were retrieved. The first (R2Bgm) is 3485 bp long, excluding the poly-(A) tail. Its ORF appears as 3164 bp long but, once again, it has one frame shift mutation disrupting the sequence at 875 bp. The second element is 3059 bp long, excluding the poly-(A) tail and it is nearly identical to R2Bgm (0.4% of divergence), with the exception of a 426 bp deletion located between positions 1035–1460. It has been therefore called R2Bgm^*del*^. This element shows a complete ORF of 2739 bp encoding a protein of 912 amino acids. Interestingly, in a 5′ end sequence survey carried out in *B. rossius*^34^, an incomplete element was retrieved (R2Br^*del*^), showing 99% of similarity with R2Bgm^*del*^ and sharing the same internal deletion. The R2Br^*del*^ fragment encompasses the whole 5′ UTR and the first 1293 bp of the ORF; the protein sequence obtained from that part of the ORF is 430 amino acids long, and the RT domain was only partially covered.

All isolated elements showed one zinc finger motif (CCHH) at the protein N-terminal end, with the exception of R2Bgg^B^ that shows two zinc finger motifs (CCHC + CCHH).

Subsequent comparisons involved presently isolated elements (*B. atticus*: R2Ba; *B. g. grandii*: R2Bgg^A^ and R2Bgg^B^; *B. g. benazzii*: R2Bgb; *B. g. maretimi*: R2Bgm and R2Bgm^*del*^ Fig. 1) together with previously identified ones (*B. rossius*: R2Br^*fun*^, R2Br^*deg*^ and R2Br^*del*^ 32,34).

The comparisons between all *Bacillus* R2 lineages at the nucleotide level indicated that R2Bgg^B^ element is the most differentiated, its divergence ranging from 55.1% (R2Bgg^A^) to 56.3% (R2Bgm^*del*^; Table 1). R2s from *B. g. maretimi* and *B. g. benazzii* are less divergent from *B. rossius* R2Br^*fun*^ and R2Br^*deg*^(3.9–4.6% and 10.3–10.6%, respectively) than from *B. g. grandii* R2Bgg^A^ and *B. atticus* R2Ba (19.3–19.6% and 19.7–20.1%, respectively; Table 1). Moreover, the R2Br^*del*^ fragment divergence ranges from 0.9% (R2Bgm) to 55.5% (R2Bgg^B^).

### Phylogenetic analyses

**Figure 2:**
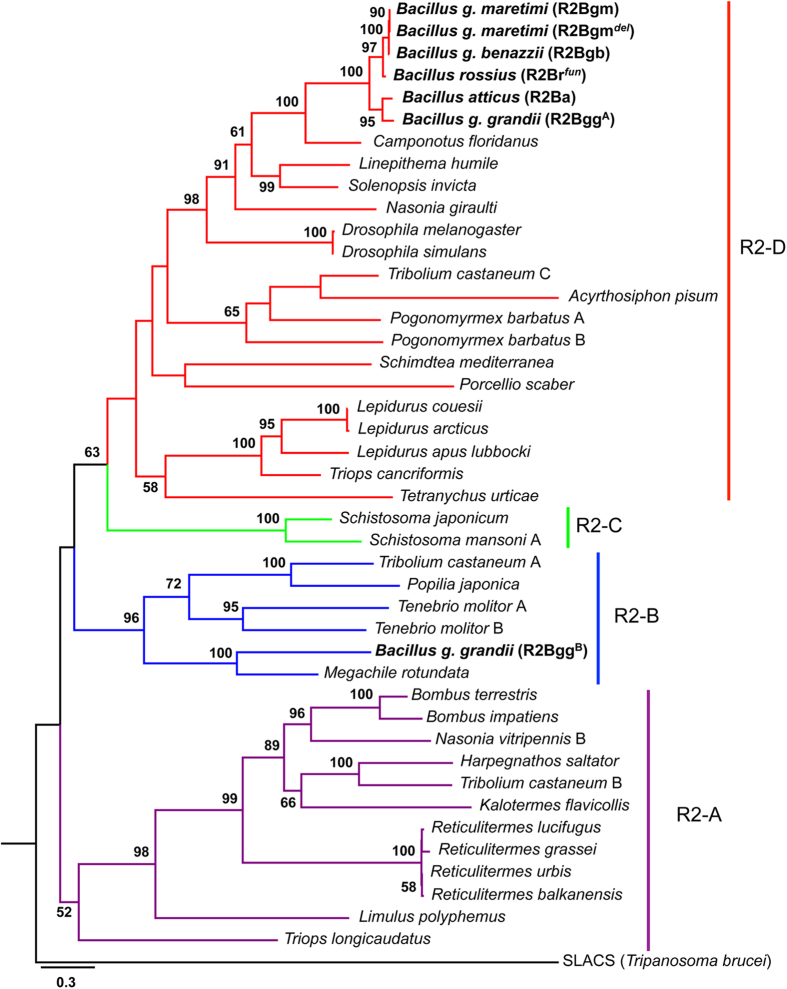
Phylogenetic position of *Bacillus* R2 elements. Maximum Likelihood tree (-ln L = 35666.30) built on RT amino acid sequence of *Bacillus* elements and of elements representative of the four main clades (R2-A, R2-B, R2-C and R2-D). Numbers at nodes represent bootstrap values ≥50%.

The Maximum Likelihood tree (Fig. 2) computed on amino acid sequences spanning from the RT domain to the C-terminal end, in addition to the RLE domain, clearly reflects the clustering pattern based on the number of zinc-finger motifs at the N-terminal end^22^,^23^. Accordingly, the element R2Bgg^B^ clusters within clade R2-B, while the remaining *Bacillus* R2s fall within the clade R2-D and are all grouped in a fully supported, monophyletic clade.

**Figure 3:**
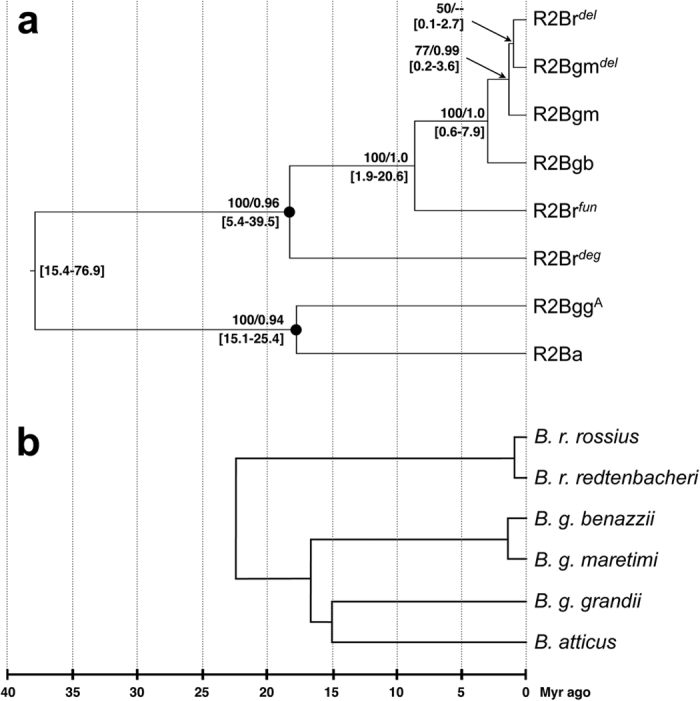
*Bacillus* R2 elements phylogeny. (**a**) Dated phylogeny based on R2 nucleotide sequences; numbers at nodes indicate, above, Maximum Likelihood (-ln L = 2449.07) bootstrap values ≥50%/Bayesian posterior probabilities ≥0.90 and, below, age estimate 95% high posterior density interval. Nodes with black dots are those where age calibration was applied. The R2Bgg^B^ element was not included due to its extreme nucleotide divergence. (**b**) Schematic drawing of *Bacillus* taxa phylogeny as derived from ref. 27.

Since amino acid sequence is not available for the degenerate element R2Br^*deg*^ and only the N-terminal end is available for R2Br^*del*^, another phylogenetic tree was constructed based on the nucleotide sequence of *Bacillus* elements only, excluding R2Bgg^B^ as its sequence is too divergent and belongs to a distantly related clade. The Maximum Likelihood and Bayesian dated phylogenies fully agree (Fig. 3a) and overlap the topology of the *Bacillus* cluster in the amino acid sequences analysis (Fig. 2). Elements from *B. g. grandii* (R2Bgg^A^) and *B. atticus* show a closer relationship while those from *B. rossius, B. g. benazzii* and *B. g. maretimi* are included in the same clade. Interestingly, *B. rossius, B. g. benazzii* and *B. g. maretimi* elements show a paraphyletic relationship, where R2s from *B. g. benazzii* and *B. g. maretimi* are grouped in a monophyletic cluster. R2Bgm^*del*^and R2Br^*del*^ show a well-supported relationship with R2Bgm. Node ages indicate that *B. rossius, B. g. benazzii* and *B. g. maretimi* elements radiated about 18 Myr ago and that the cluster including *B. g. benazzii* and *B. g. maretimi* R2s split from R2Br^*fun*^ around 8.6 Myr ago. Finally, R2Bgb and R2Bgm/R2Bgm^*del*^/R2Br^*del*^ diverged about 2.6 Myr ago.

The R2 tree topology is only partially congruent with the host species phylogeny (Fig. 3b), the significant difference being the placement of *B. g. benazzii* and *B. g. maretimi.* In fact, these two taxa are known to have closer relationship with *B. g. grandii* and *B. atticus* than with *B. rossius*^26^,^27^.

### Divergence vs. age analysis

**Figure 4:**
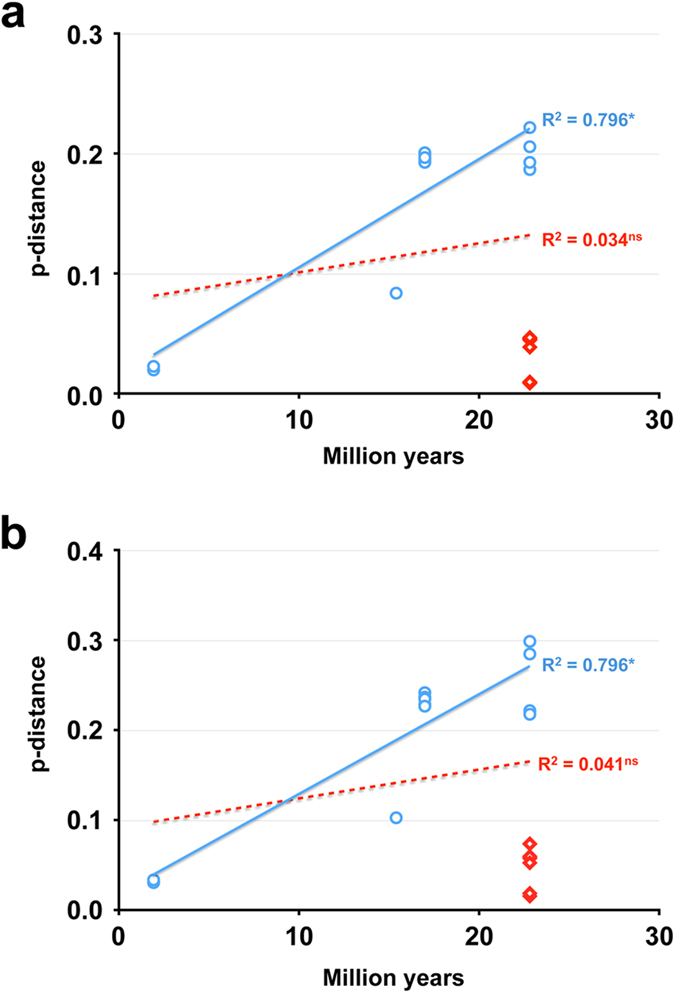
Age *versus* divergence analysis. Plot of host age *vs*. nucleotide divergence (**a**) and *vs*. amino acid divergence (**b**). Red diamonds indicate comparisons relative to putative horizontal transfers (HTs). Two trend-lines are reported: the dotted one is relative to the analysis including all comparisons; the filled one is relative to the analysis that exclude putative HT comparisons. Correlation coefficients (R^2^), together with probability (ns: not significant; *p < 0.001), are reported near the respective trend-line.

When the nucleotide or amino acid sequence divergences were plotted against host species split ages, comparisons between R2 *B. rossius* (R2Br*fun*, R2Br^*del*^), *B. g. benazzii* (R2Bgb) and *B. g. maretimi* (R2Bgm, R2Bgm^*del*^) elements were found less divergent than expected (Fig. 4). In fact, they showed nucleotide p-distance values ranging from 0.009 (R2Bgm *vs*. R2Br^*del*^) to 0.047 (R2Bgb vs. R2Br^*del*^), while co-eval sequence divergences ranged from 0.187 (R2Bgg *vs*. R2Br*fun*) to 0.222 (R2Bgg *vs*. R2Br^*del*^) (Table 1; Fig. 4a). Amino acid sequence divergences followed the same trend (Table 1; Fig. 4b). Interestingly, when all comparisons were plotted R^2^ values resulted very low (0.034 and 0.041 for nucleotide and amino acid analyses, respectively) and correlations were not significant. On the other hand, when comparisons between R2 *B. rossius, B. g. benazzii* and *B. g. maretimi* elements were excluded, R^2^ values resulted higher (0.796 for both nucleotide and amino acid analyses) and correlations became significant (p < 0.001; Fig. 4).

## Discussion

R2 non-LTR elements are probably among the most widely studied TEs in both model and non-model organisms. One of the main issues regarding R2 evolution is the frequent incongruence between its phylogeny and that of the host species, for which different hypotheses have been put forward. Kojima and Fujiwara^22^ while analyzing a number of elements sampled across metazoan, observed numerous instances of “local” congruence between R2 and host phylogenies and did not identify clear cases of putative HT. Instead, they found instances of paralogous lineages. Therefore, they concluded that the evolution of R2 can be explained by vertical inheritance with extensive paralogous lineages extinction/diversification. Luchetti and Mantovani^23^, in addition, suggested that in some ancestral genomes a library of unrelated elements might have been present. The differential amplification of these elements in the derived taxa - irrespective of their phylogeny - would also explain the presence in some genomes of multiple, unrelated R2 lineages.

Data reported here, though, suggest the possibility of HT between congeneric species of stick insects. In order to verify this aspect, we checked if data fit the three criteria that are considered relevant to identify an HT event: (i) a divergence between elements from different species lower than expected on the basis of host split ages, (ii) the phylogenetic incongruence between the putatively transferred element and the host species, and (iii) the patchy distribution among closely related taxa^3^,^5^.

R2 elements of *B. g. benazzii* and *B. g. maretimi* are less divergent from *B. rossius* R2Br^*fun*^ than from the element of the conspecific *B. g. grandii*. Accordingly, the divergence *versus* age analysis indicated the comparisons R2Bgb/R2Br^*fun*^, R2Bgb/R2Br^*del*^, R2Bgm/R2Br^*fun*^, R2Bgm/R2Br^*del*^, R2Bgm^*del*^/R2Br^*fun*^and R2Bgm^*del*^/R2Br^*del*^ as significantly less divergent than expected on the basis of the host split age (22.8 Myr ago)^27^.

The phylogenetic analyses conducted on amino acid sequences indicated that all *Bacillus* elements have a well-supported monophyletic origin and fall in the R2-D clade, with the exception of the R2Bgg^B^ element that is nested within the R2-B clade. This distribution is in full agreement with the number and type of zinc-finger motifs found at N-terminal end^22^,^23^. Both phylogenetic analyses conducted on amino acid and nucleotide sequence evidenced that, within the *Bacillus* cluster in the R2-D clade, the relationships between elements only partially overlap those between host taxa. In fact, elements from *B. g. maretimi, B. g. benazzii* and *B. rossius* group together in a well-supported cluster in clear-cut contrast with the sister relationship occurring between the *B. g. maretimi*/*B. g. benazzii* clade and the *B. g. grandii*/*B. atticus* one, evidenced on the basis of allozymes, mitochondrial DNA and satellite DNA^26^,^27^.

Patchy distribution refers to the presence of a given element in one lineage and its absence within the sister lineage. In the case of R2, beside the number of zinc fingers, there are no unequivocal criteria to determine lineages; on the other hand, Stage and Eickbush^35^, in *Drosophila* species, considered that elements diverging >1% would represent separated lineages. If we take into account this threshold, then R2Bgm^*del*^ and R2Br^*del*^ belong to the same lineage (but not to R2Bgm due to the large deletion occurring within the ORF). Therefore, the “R2Bgm^*del*^-R2Br^*del*^” lineage shows a patchy distribution.

On the whole, data presented here suggest two interspecific HT events in *Bacillus* stick insects, one occurring from *B. rossius* toward the *B. g. benazzii*/*B. g. maretimi* ancestor, accounting for R2Bgb and R2Bgm elements origin from R2Br^*fun*^, and another one from *B. g. maretimi* toward *B. rossius,* accounting for the origin of R2Br^*del*^ from R2Bgm^*del*^element (Supplementary Figure S1). Random lineage loss and/or the possibility of having missed some lineages in *B. g. grandii* and *B. atticus* could explain the patchy distribution and phylogenetic incongruence: however, the observed divergence, which is lower than the expected one considering the host species split, appears to rule out these possibilities. In addition, notwithstanding the large error associated with age estimates, dated phylogeny clearly indicates a more recent origin of the R2s involved in HTs with respect to species/subspecies split age. In R2 phylogeny, the *Bacillus* lineages including R2Bgb and R2Bgm derived from the R2Br^*fun*^ element with a divergence time of about 8.6 Myr ago; in contrast, *B. rossius* and *B. grandii* taxa split more than 22 Myr ago^27^. Moreover, the monophyletic origin of R2Bgm, R2Bgm^*del*^and R2Br^*del*^ and their very recent radiation (less than 1 Myr ago) suggest that the second HT event would have happened much more recently.

TE horizontal transfer has been linked to vectors able to survive outside the host cell (for example virus)^16^,^36^, or to host-parasite^14^ or predator-prey^17^ relationships. Actually, we do not have evidences that may explain the HT between *Bacillus* taxa following these routes, although a parasite-mediated transfer cannot be disregarded: *Bacillus* stick insects, in fact, have been recently observed to be infected by nematodes without apparent loss of fecundity (Mantovani, personal observation).

HT has also been attributed to hybridization/introgression^18^,^19^ and R2 HT events can be well explained taking into consideration *Bacillus* parental and hybrid taxa reproductive biology which “provides unusual opportunities for genomes to cycle between the various systems”^37^. In particular, *B. rossius* and *B. g. benazzii* are the maternal and paternal species, respectively, of the hybridogenetic strain *B. rossius* - *g. benazzii*: these are hybrid females expressing both parental haplosets in the somatic tissues, but they discard the paternal *B. g. benazzii* haploset in the germ line. Before meiosis, the paternal haploset is eliminated and the maternal one is duplicated; thus, meiosis involves two maternal haplosets. This prevents the recombination between paternal and maternal chromosomes^26^. Accordingly, extensive allozyme data obtained from the hybridogenetic system have never retrieved the occurrence of recombination between the maternal and paternal haplosets^28^,^38^. Then, the egg, containing the unrecombined *B. rossius* maternal haploset, can be fertilized by a sperm from a *B. g. benazzii* male, restoring the hybrid condition; in the lab, hybridogenesis occur also with *B. g. maretimi* and *B. rossius* males; in the latter instance, pure *B. rossius* individuals are produced (Supplementary Figure S2)^25^,^26^,^37^. However, among hybridogenetic descendants, instances of maternal haploset exclusion at meiosis or of androgenesis may occur (Supplementary Figure S2)^26^,^28^,^38^. Maternal *B. rossius* haploset exclusion leads to an egg containing the paternal *B. g. benazzii* haploset: through the paternal haploset doubling at the development onset, a diploid *B. g. benazzii* nuclear genome in a *B. rossius* cytoplasm can be reconstituted. It is to be noted, though, that this instance has been only rarely observed^28^,^38^ and that the same kind of offspring could be obviously obtained through a backcross with a *B. g. benazzii* male. In *Bacillus* stick insects, androgenesis takes place when the loss of both parental genomes from the egg nucleus is followed by the entrance and mixes of two sperm nuclei. This reconstitutes a diploid genome, resulting in a fertile offspring with a fully-paternal nuclear genome and a maternal cytoplasm. Breeding experiments in lab populations resulted in a high number of androgenetic individuals, especially when crossing either *B. g. maretimi* or *B. rossius* males.

**Figure 5:**
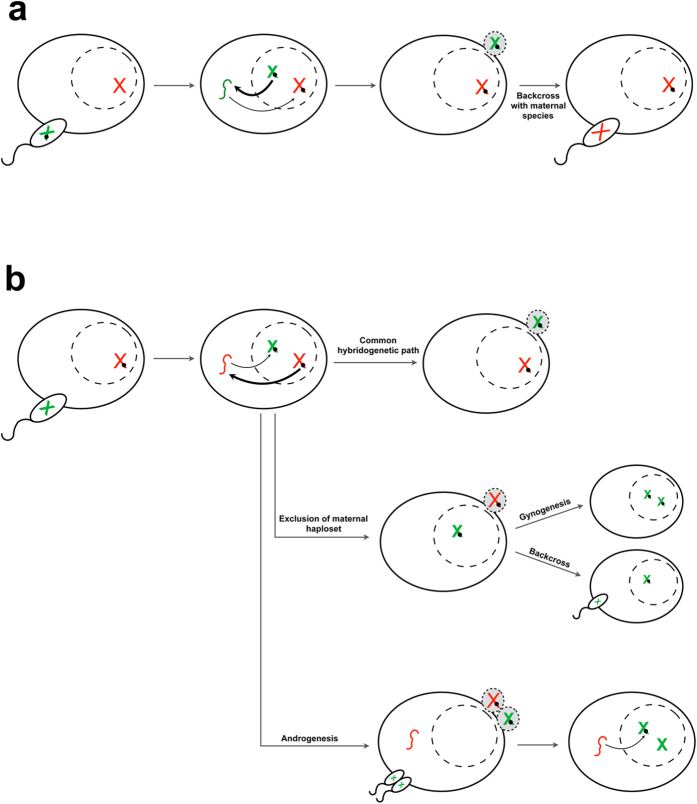
Possible models of R2 horizontal transfer (HT) in *Bacillus* stick insects. (**a**) HT of an element from the paternal to the maternal species haploset. Via a standard hybridogenetic path followed by a backcross with a male of the maternal species, the element can invade the genome of the maternal species. (**b**) Horizontal transfer of an element from the maternal to the paternal species haploset. Following the standard hybridogenetic path, the element has no chance to enter into the paternal species genome given that the paternal haploset carrying the transferred element is lost. Only instances of maternal haploset exclusion may lead to offspring with fully paternal genome either through gynogenesis or backcross. Also androgenesis produces offspring with fully paternal genome in a maternal cytoplasm. In this case, though, the HT event must be mediated by the element RNA intermediate that allows the transfer from the excluded hybridogenetic genomes to the one derived from two sperm nuclei mixis. Dashed circles within cells are nuclei; red chromosomes represent the maternal haploset and green chromosomes represent the paternal haploset. Red and green small lines within cell cytoplasm represent transcribed RNAs. Thick and thin arrows indicate transcription and re-integration, respectively. Excluded haplosets are shaded in grey.

In this context, HT events described here may have followed different routes. The common hybridogenetic pattern nicely explains the case of an element originating in the paternal genome and transferred to the maternal one. During the hybridogenetic stage, retrotransposition may lead to insertion in the maternal haploset; therefore, when the egg is fertilized by a maternal species male, the R2 element may enter into the maternal species lineage in the following generations (Fig. 5a). Thus, the HT involving R2Bgm^*del*^ and R2Br^*del*^ could have followed this route less than 2 Myr ago. *B. g. maretimi* diverged from *B. g. benazzii* about 2 Myr ago and it is presently restricted to the Marettimo Island. Although we have no evidence at present of *B. rossius* - *B. g. maretimi* hybridogenesis in nature, populations of the two taxa may have come into contact during the Pliocenic-Pleistocenic marine transgression that have connected the Island of Marettimo to western Sicily, allowing hybridization.

On the other hand, the interspecific transfer of an element from the maternal haploset to the paternal one, as in the case of R2Br^*fun*^giving origin to the R2Bgm/R2Bgb lineage, cannot be explained by an hybridogenetic route (first example of Fig. 5b). This could have occurred by one of the two possible patterns following the maternal haploset exclusion (second example of Fig. 5b) or via androgenesis. In the latter instance, no inheritance of any part of the hybrid nuclear genome is observed, as the offspring genome is given by the haplosets of two additional sperms. On the other hand, the androgenetic offspring inherits from the maternal species the cytoplasm. Here R2 RNAs can be present as, after transcription and maturation, they are transferred from the nucleus to the cytoplasm for proteins translations. RNAs, then, re-enter into the nucleus for the integration process (Supplementary Figure S3)^39^. It is thus possible that cytoplasmic R2 RNAs re-entered into the nucleus and re-integrated after the mixis of two sperms’ nuclei (third example in Fig. 5b). As suggested by data, the HT event originating both R2Bgb and R2Bgm was a single event, dating back to 7 Myr ago, while R2Bgb and R2Bgm divergence time was close to that of the pertaining *B. grandii* subspecies split (~2 Myr ago, timing actually overlapping if we consider the error associated to the node age estimate). At variance of the recent split of *B. g. benazzii* and *B. g. maretimi* subspecies, *B. rossius* populations carrying R2Br^*fun*^ were already present before the end of the Messinian (~5.4 Myr ago)^27^,^32^. Following these evidences, we can hypothesize that hybridization may have already taken place between *B. rossius* and the ancestor of *B. g. benazzii*/*B. g. maretimi* and, in this scenario, the element R2Br^*fun*^ could have been transferred into the *B. g. benazzii*/*B. g. maretimi* ancestor’s genome. It is finally to be noted that, with the exception of the derived R2Bgm^*del*^, we did not detect other R2 lineages in *B. g. benazzii* nor in *B. g. maretimi*; it is thus likely that the invasion of the new R2 element led to the replacement of other possible R2 lineage(s) present in the ancestor genome.

On the whole, analyses presented here support the occurrence of HTs involving R2 retrotransposons, a process previously undetected for this element on the basis of a metazoan-wide analysis^22^. The peculiar way by which HT events seem to have occurred in stick insects further suggests some interesting speculations. Introgression is commonly observed when inter-specific hybridization occurs, and it is thought to bear beneficial effect on the receiving genome by promoting adaptation^40^. In *Bacillus* hybridogenetic lineages, though, gene introgression has never been observed possibly owing to the peculiar cytological mechanism of haploset exclusion during egg maturation that prevents recombination^41^, but as above suggested TE spreading may not require crossing over events.

Transposons are well-known promoters of genome evolution: therefore, possible HTs not only would allow TE survival but may also bring this advantage to the invaded genome. In this view, the role of hybridization as a contributor to genome and species evolution^39^,^41^ can be achieved also by mean of TEs transfer from one genome to another, instead of a classical introgression. It is to be noted, in fact, that successful introgression usually occurs after several round of hybridization/backcrossing^42^ while a TEs HT may even require a single generation.

Taking into account the complex history of hybridization and intertaxa relationships that characterize *Bacillus* stick insects, they can constitute an interesting evolutionary framework in which to address studies of such phenomena.

## Materials and Methods

### Sampling, DNA isolation and R2 sequencing

Samples of the obligatory parthenogenetic *Bacillus atticus* (subsp. *atticus*) and of the three subspecies of the strictly gonochoric *Bacillus grandii* were field-collected in Sicily and immediately frozen at −80 °C. Full-length R2 elements were isolated and characterized from one female of *B. atticus* (Scoglitti; BattSCO♀25) and from one male each of *B. g. grandii* (Ponte Manghisi; BggPMA♂54), *B. g. benazzii* (Torre Bennistra; BgbTBE♂4) and *B. g. maretimi* (Marettimo Island; BgmMAR♂2).

Total DNA was extracted from single stick insect legs with the standard phenol/chloroform protocol. R2 was isolated through PCR amplification, cloning and sequencing^32^. Universal and specifically designed primers used in this study are reported in Supplementary Table S1.

### Sequence analysis

Sequences were edited and assembled using MEGA v. 6.0^43^ and open reading frames (ORFs) were searched with the ORF Finder tool server (available at: http://www.ncbi.nlm.nih.gov/gorf/gorf.html). All newly characterized R2 elements are reported as Supplementary Material S1. Nucleotide and amino acid sequence alignments have been carried out using MAFFT 7.2^44^ with L-INS-i parameters. Sequence divergences, calculated as uncorrected *p-*distances, were obtained using MEGA v. 6.0.

### Phylogenetic analyses

Presently obtained R2 elements have been analyzed together with those previously isolated from *B. rossius*: the functional element R2Br^*fun*^ and the degenerate one R2Br^*deg*^ 32. Present analysis includes also the 5′ half of a new element, carrying an internal deletion (hence called R2Br^*del*^) obtained from *B. rossius* while screening for the 5′ end sequence variation^34^.

Two phylogenetic analyses have been performed. The first analysis was based on inferred amino acid sequences encompassing the reverse transcriptase (RT) and the restriction enzyme-like endonuclease (RLE) domains. This analysis included elements from several metazoan representatives of the main R2 clades (Supplementary Table S2)^22^,^23^,^45^,^46^,^47^,^48^,^49^,^50^,^51^,^52^. The second analysis included only *Bacillus* elements (with the exception of R2Bgg^B^) and was based on nucleotide sequences.

The best substitution models, chosen on the basis of the Bayesian information criterion (BIC), were calculated as LG + G + I for amino acid dataset using Prottest v. 3.4^53^ and as GTR + G for nucleotide dataset with jModelTest v. 2.1.7^54^. Maximum Likelihood phylogenetic analyses, with 100 bootstrap replicates for nodal support, have been carried out using PhyML v. 3.0^55^. A dated phylogeny has been also build on the nucleotide sequence dataset using a Bayesian analysis implemented in BEAST v. 1.8^56^. Two runs were performed with 3 × 10^7^ generations and evaluated for convergence both graphically and checking for Estimated Sample Size (ESS) > 200. The tree search was setup using an uncorrelated, log-normal relaxed molecular clock and the Birth-Death speciation process. This analysis produces a tree topology with nodal supports (posterior probabilities) and relative node age estimates. Age calibration was implemented using secondary calibration points obtained from the *Bacillus* species dated phylogeny^27^. We calibrated the divergence between the *B. rossius* functional (R2Br^*fun*^) and degenerate (R2Br^*deg*^) elements^32^ at a minimum of 5.4 Myr ago, that is the split between European *B. r. rossius*/*B. r. redtenbacheri* and the North African *B. r. tripolitanus* A: as all these taxa retain the two elements^32^, R2s divergence should date before their subspecific split. Prior distribution of this calibration was modelled using an uniform distribution with hard bounds and a maximum of 50 Myr ago. We also calibrated the divergence between R2Bgg^A^ and R2Ba (for R2 lineages acronyms see Fig. 1) based on the estimated split age between *B. g. grandii* and *B. atticus* at ~15.4 Myr ago. Prior distribution of this calibration was modelled using a log-normal distribution, with soft bounds (maximum set at 50 Myr ago), as there is little probability of a younger R2 divergence but, since multiple R2 lineages can be commonly found in a genome, it is possible that they may have diverged before the host species split.

A divergence *versus* age analysis has been performed by plotting nucleotide and amino acid sequence divergences against the host age split. This analysis is based on the principle that elements deriving from a HT are less divergent than expected from the host-split age, while paralogous lineages are more divergent than expected^3^,^5^,^8^.

## Additional Information

**How to cite this article:** Scavariello, C. *et al*. Hybridogenesis and a potential case of R2 non-LTR retrotransposon horizontal transmission in *Bacillus* stick insects (Insecta Phasmida). *Sci. Rep.* **7**, 41946; doi: 10.1038/srep41946 (2017).

**Publisher's note:** Springer Nature remains neutral with regard to jurisdictional claims in published maps and institutional affiliations.

## Supplementary Material

Supplementary Information

## Figures and Tables

**Table 1:** Nucleotide and amino acid (in parentheses) p-distances between *Bacillus* R2 elements (below the diagonal).

	1	2	3	4	5	6	7	8	9
**1. R2Ba**		0.005 (0.009)	0.009 (0.014)	0.007 (0.013)	0.007 (0.013)	0.007 (0.014)	0.007 (0.013)	0.007 (n/c)	0.011 (0.022)
**2. R2Bgg**^**A**^	0.084 (0.103)		0.009 (0.014)	0.007 (0.013)	0.007 (0.013)	0.007 (0.014)	0.007 (0.013)	0.007 (n/c)	0.011 (0.022)
**3. R2Bgg**^**B**^	0.555 (0.731)	0.551 (0.731)		0.009 (0.014)	0.009 (0.014)	0.009 (0.015)	0.009 (0.014)	0.009 (n/c)	0.013 (0.021)
**4. R2Bgb**	0.197 (0.227)	0.193 (0.237)	0.559 (0.732)		0.002 (0.005)	0.002 (0.006)	0.004 (0.007)	0.005 (n/c)	0.005 (0.013)
**5. R2Bgm**	0.199 (0.229)	0.195 (0.235)	0.559 (0.733)	0.020 (0.031)		0.001 (0.002)	0.004 (0.007)	0.005 (n/c)	0.002 (0.006)
**6. R2Bgm**^***del***^	0.201 (0.235)	0.196 (0.242)	0.563 (0.737)	0.023 (0.034)	0.004 (0.004)		0.004 (0.008)	0.006 (n/c)	0.003 (0.007)
**7. R2Br**^***fun***^	0.193 (0.218)	0.187 (0.222)	0.557 (0.730)	0.039 (0.053)	0.045 (0.058)	0.046 (0.060)		0.005 (n/c)	0.006 (0.012)
**8. R2Br**^***deg***^	0.200 (n/c)	0.197 (n/c)	0.560 (n/c)	0.103 (n/c)	0.106 (n/c)	0.106 (n/c)	0.094 (n/c)		0.009 (n/c)
**9. R2Br**^***del***^	0.206 (0.285)	0.222 (0.299)	0.555 (0.756)	0.047 (0.074)	0.009 (0.016)	0.010 (0.019)	0.056 (0.072)	0.124 (n/c)	

Standard errors are shown above the diagonal. Amino acid distances for R2Br^*deg*^ has not been calculated (n/c) because of the high degeneracy of the ORF. Acronyms are as in Fig. 1.

## References

[b1] Chénais B., Caruso A., Hiard S. & Casse N. The impact of transposable elements on eukaryotic genomes: from genome size increase to genetic adaptation to stressful environments. Gene 509(1), 7–15 (2012). 22921893 10.1016/j.gene.2012.07.042

[b2] Fujiwara H. Site specific non-LTR retrotransposons. *Microbiol*. Spectr. 3(2), MDNA3-0001-2014 (2015). 10.1128/microbiolspec.MDNA3-0001-2014 26104700

[b3] Loreto E. L. S., Carareto C. M. & Capy P. Revisiting horizontal transfer of transposable elements in *Drosophila*. Heredity 100, 545–554 (2008). 18431403 10.1038/sj.hdy.6801094

[b4] Schaack S., Gilbert C. & Feschotte C. Promiscuous DNA: horizontal transfer of transposable elements and why it matters for eukaryotic evolution. Trends Ecol. Evol. 25, 537–546 (2010). 20591532 10.1016/j.tree.2010.06.001 PMC2940939

[b5] Silva J. C., Loreto E. L. & Clark J. B. Factors that affect the horizontal transfer of transposable elements. Curr. Issues Mol. Biol. 6, 57–71 (2004). 14632259

[b6] Malik H. S., Henikoff S. & Eickbush T. H. Poised for contagion: Evolutionary origins of the infectious abilities of invertebrate retroviruses. Genome Res. 10, 1307–1318 (2000). 10984449 10.1101/gr.145000

[b7] Eickbush D. G. & Eickbush T. H. Vertical transmission of the retrotransposable elements R1 and R2 during the evolution of the *Drosophila melanogaster* species subgroup. Genetics 139, 671–684 (1995). 7713424 10.1093/genetics/139.2.671 PMC1206373

[b8] Malik H. S., Burke W. D. & Eickbush T. H. The Age and Evolution of Non-LTR Retrotransposable Elements. Mol. Biol. Evol. 16(6), 793–805 (1999). 10368957 10.1093/oxfordjournals.molbev.a026164

[b9] Novikova O., Śliwińska E., Fet V., Settele J., Blinov A. & Woyciechowski M. CR1 clade of non-LTR retrotransposons from *Maculinea* butterflies (Lepidoptera: Lycaenidae): evidence for recent horizontal transmission. BMC Evol. Biol. 7, 93 (2007). 17588269 10.1186/1471-2148-7-93 PMC1925062

[b10] Ivancevic A. M., Walsh A. M., Kortschak R. D. & Adelson D. L. Jumping the fine LINE between species: horizontal transfer of transposable elements in animals catalyses genome evolution. Bioessays 35(12), 1071–1082 (2013). 24003001 10.1002/bies.201300072

[b11] Biedler J. K., Chen X. & Tu Z. Horizontal transmission of an R4 clade non-long terminal repeat retrotransposon between the divergent *Aedes* and *Anopheles* mosquito genera. Insect Mol. Biol. 24(3), 331–337 (2015). 25615532 10.1111/imb.12160 PMC4400214

[b12] Piskurek O. & Okada N. Poxviruses as possible vectors for horizontal transfer of retroposons from reptiles to mammals. Proc. Natl. Acad. Sci. USA 104(29), 12046–12051 (2007). 17623783 10.1073/pnas.0700531104 PMC1924541

[b13] Luchetti A., Šatović E., Mantovani B. & Plohl M. RUDI, a short interspersed element of the V-SINE superfamily widespread in molluscan genomes. Mol. Genet. Genomics 291(3), 1419–1429 (2016). 26987730 10.1007/s00438-016-1194-z

[b14] Gilbert C., Schaack S., Pace J. K. II, Brindley P. J. & Feschotte C. A role for host-parasite interactions in the horizontal transfer of transposons across phyla. Nature 464, 1347–1350 (2010). 20428170 10.1038/nature08939 PMC3004126

[b15] Dupeyron M., Leclercq S., Cerveau N., Bouchon D. & Gilbert C. Horizontal transfer of transposons between and within crustaceans and insects. Mob. DNA 5(1), 4 (2014). 24472097 10.1186/1759-8753-5-4 PMC3922705

[b16] Coates B. S. Horizontal transfer of a non-autonomous Helitron among insect and viral genomes. BMC Genomics 27, 16:137 (2015). 10.1186/s12864-015-1318-6 PMC4344730 25766741

[b17] Tang Z., Zhang H. H., Huang K., Zhang X. G., Han M. J. & Zhang Z. Repeated horizontal transfers of four DNA transposons in invertebrates and bats. Mob. DNA. 6(1), 3 (2015). 25606061 10.1186/s13100-014-0033-1 PMC4298943

[b18] Kofler R., Hill T., Nolte V., Betancourt A. J. & Schlötterer C. The recent invasion of natural *Drosophila simulans* populations by the P-element. Proc. Natl. Acad. Sci. USA 112(21), 6659–6663 (2015). 25964349 10.1073/pnas.1500758112 PMC4450375

[b19] Mallet J., Besansky N. & Hahn M. W. How reticulated are species? Bioessays 38, 140–149 (2015). 26709836 10.1002/bies.201500149 PMC4813508

[b20] Eickbush T. & Malik H. Origins and evolution of retrotransposons in Mobile DNA II (eds Craig N. L., Gellert R. C. M. & Lambowitz A. M.) 1111–1144 (American Society for Microbiology Press, Washington, DC, 2002).

[b21] Kojima K. K., Kuma K., Toh H. & Fujiwara H. Identification of rDNA-specific non-LTR retrotransposons in Cnidaria. Mol. Biol. Evol. 23, 1984–1993 (2006). 16870681 10.1093/molbev/msl067

[b22] Kojima K. K. & Fujiwara H. Long-term inheritance of the 28S rDNA-specific retrotransposon R2. Mol. Biol. Evol. 22, 2157–2165 (2005). 16014872 10.1093/molbev/msi210

[b23] Luchetti A. & Mantovani B. Non-LTR R2 element evolutionary patterns: phylogenetic incongruences, rapid radiation and the maintenance of multiple lineages. Plos One 8(2), e57076 (2013). 23451148 10.1371/journal.pone.0057076 PMC3581529

[b24] Jakubczak J. L., Zenni M. K., Woodruff R. C. & Eickbush T. H. Turnover of R1 (type I) and R2 (type II) retrotransposable elements in the ribosomal DNA of *Drosophila melanogaster*. Genetics 131(1), 129–42 (1992). 1317313 10.1093/genetics/131.1.129 PMC1204947

[b25] Pérez-Gonzalez C. E. & Eickbush T. H. Dynamics of R1 and R2 elements in the rDNA locus of *Drosophila simulans*. Genetics 158, 1557–1567 (2001). 11514447 10.1093/genetics/158.4.1557 PMC1461747

[b26] Scali V., Passamonti M., Marescalchi O. & Mantovani B. Linkage between sexual and asexual lineages: genome evolution in *Bacillus* sticks insects. Biol. J. Linn. Soc. 79, 137–150 (2003).

[b27] Mantovani B., Passamonti M. & Scali V. The mitochondrial cytochrome oxidase II gene in *Bacillus* stick insects: ancestry of hybrids, androgenesis, and phylogenetic relationships. Mol. Phylogenet. Evol. 19, 157–163 (2001). 11286500 10.1006/mpev.2000.0850

[b28] Mantovani B. & Scali V. Hybridogenesis and androgenesis in the stick-insect *Bacillus rossius-grandii benazzii* (Insecta Phasmatodea). Evolution 46(3), 783–796 (1992). 10.1111/j.1558-5646.1992.tb02084.x 28568678

[b29] Lehtonen J., Schmidt D. J., Heubel K. & Kokko H. Evolutionary and ecological implications of sexual parasitism. Trends Ecol. Evol. 28(5), 297–306 (2013). 23399316 10.1016/j.tree.2012.12.006

[b30] Darras H., Leniaud L. & Aron S. Large-scale distribution of hybridogenetic lineages in a Spanish desert ant. Proc. R. Soc. B 281, 20132396 (2014). 10.1098/rspb.2013.2396 PMC3843834 24225458

[b31] Avise J. C. Evolutionary perspectives on clonal reproduction in vertebrate animals. Proc. Natl. Acad. Sci. USA 112, 8867–8873 (2015). 26195735 10.1073/pnas.1501820112 PMC4517198

[b32] Bonandin L., Scavariello C., Luchetti A. & Mantovani B. Evolutionary dynamics of R2 retroelement and insertion inheritance in the genome of bisexual and parthenogenetic *Bacillus rossius* populations (Insecta, Phasmida). Insect Mol. Biol. 23(6), 808–820 (2014). 25134735 10.1111/imb.12126

[b33] Bonandin L., Scavariello C., Mingazzini V., Luchetti A. & Mantovani B. Obligatory parthenogenesis and TE load: *Bacillus* stick insects and the R2 non-LTR retrotransposon. Insect Sci. doi: 10.1111/1744-7917.12322 (2016). 26813995

[b34] Martoni F., Eickbush D. G., Scavariello C., Luchetti A. & Mantovani B. Dead element replicating: degenerate R2 element replication and rDNA genomic turnover in the *Bacillus rossius* stick insect (Insecta: Phasmida). Plos One 10, e0121831 (2015). 25799008 10.1371/journal.pone.0121831 PMC4370867

[b35] Stage D. E. & Eickbush T. H. Origin of nascent lineages and the mechanisms used to prime second-strand DNA synthesis in the R1 and R2 retrotransposons of *Drosophila*. Genome Biol. 10(5), R49 (2009). 19416522 10.1186/gb-2009-10-5-r49 PMC2718515

[b36] Piskurek O. & Okada N. Tracking the ancestry of a deeply conserved eumetazoan SINE domain. Mol. Biol. Evol. 28(10), 2727–2730 (2011). 21512106 10.1093/molbev/msr115

[b37] Burt A. & Trivers R. L. Genes in Conflict (Belknap Press of Harvard University Press, Boston, MA, 2006).

[b38] Tinti F., Mantovani B. & Scali V. Reproductive features of homospecific hybridogenetically derived stick insects suggest how unisexuals can evolve. J. evol. Biol. 8, 81–92 (1995).

[b39] Han J. S. & Boeke J. D. LINE-1 retrotransposons: modulators of quantity and quality of mammalian gene expression? Bioessays 27, 775–784 (2005). 16015595 10.1002/bies.20257

[b40] Mallet J. Hybridization as an invasion of the genome. Trends Ecol. Evol. 20, 229–237 (2005). 16701374 10.1016/j.tree.2005.02.010

[b41] Scali V., Tinti F., Mantovani B. & Marescalchi O. Mate recognition and gamete cytology features allow hybrid species production and evolution in *Bacillus* stick insects. Bollettino di Zoologia 62(1), 59–70 (1995).

[b42] Baack E. J. & Rieseberg L. H. A genomic view of introgression and hybrid speciation. Curr. Opin. Genetics Dev. 17, 513–518 (2007). 10.1016/j.gde.2007.09.001 PMC2173880 17933508

[b43] Tamura K., Stecher G., Peterson D., Filipski A. & Kumar S. MEGA6: Molecular Evolutionary Genetics Analysis version 6.0. Mol Biol Evol. 30(12), 2725–2729 (2013). 24132122 10.1093/molbev/mst197 PMC3840312

[b44] Katoh K. & Standley D. M. MAFFT Multiple Sequence Alignment Software Version 7: Improvements in Performance and Usability. Mol. Biol. Evol. 30, 772–780 (2013). 23329690 10.1093/molbev/mst010 PMC3603318

[b45] Mingazzini V., Luchetti A. & Mantovani B. R2 dynamics in *Triops cancriformis* (Bosc, 1801) (Crustacea, Branchiopoda, Notostraca): turnover rate and 28S concerted evolution. Heredity 106, 567–575 (2011). 20628416 10.1038/hdy.2010.86 PMC3183910

[b46] Mingazzini V. Transposable elements: structure and dynamic of the autonomous retrotransposon R2 in Arthropoda with non-canonical reproduction. Ph. D. thesis, University of Bologna (2011).

[b47] Ghesini S., Luchetti A., Marini M. & Mantovani M. The non-LTR retrotransposon R2 in termites (Insecta, Isoptera): characterization and dynamics. J. Mol. Evol. 72, 296–305 (2011). 21259002 10.1007/s00239-011-9430-y

[b48] Luchetti A., Mingazzini V. & Mantovani B. 28S junctions and chimeric elements of the rDNA targeting non LTR retrotransposon R2 in crustacean living fossils (Brachiopoda, Notostraca). Genomics 100, 51–56 (2012). 22564473 10.1016/j.ygeno.2012.04.005

[b49] Burke W. D., Malik H. S., Jones J. P. & Eickbush T. H. The domain structure and retrotransposition mechanism of R2 elements are conserved throughout arthropods. Mol. Biol. Evol. 16, 502–511 (1999). 10331276 10.1093/oxfordjournals.molbev.a026132

[b50] Stage D. E. & Eickbush T. H. Maintenance of multiple lineages of R1 and R2 retrotransposable elements in the ribosomal RNA gene loci of *Nasonia*. Insect Mol Biol. 19 Suppl. 1, 37–48 (2010). 20167016 10.1111/j.1365-2583.2009.00949.x

[b51] Burke W. D., Eickbush D. G., Xiong Y., Jakubczak J. & Eickbush T. H. Sequence relationship of retrotransposable elements R1 and R2 within and between divergent insect species. Mol. Biol. Evol. 10, 163–185 (1993). 8383793 10.1093/oxfordjournals.molbev.a039990

[b52] Burke W. D., Malik H. S., Lathe W. C. & Eickbush T. H. Are retrotransposons long-term hitchhikers? Nature 392, 141–142 (1998). 9515960 10.1038/32330

[b53] Darriba D., Taboada G. L., Doallo R. & Posada D. ProtTest 3: fast selection of best-fit models of protein evolution. Bioinformatics 27, 1164–1165 (2011). 21335321 10.1093/bioinformatics/btr088 PMC5215816

[b54] Darriba D., Taboada G. L., Doallo R. & Posada D. jModelTest 2: more models, new heuristics and parallel computing. Nat. Methods 9, 772 (2012). 10.1038/nmeth.2109 PMC4594756 22847109

[b55] Guindon S., Dufayard J. F., Lefort V., Anisimova M., Hordijk W. & Gascuel O. New algorithms and methods to estimate maximum-likelihood phylogenies: assessing the performance of PhyML 3.0. Syst. Biol. 59(3), 307–321 (2010). 20525638 10.1093/sysbio/syq010

[b56] Drummond A. J. & Rambaut A. BEAST: Bayesian evolutionary analysis by sampling trees. BMC Evol. Biol. 7, 214 (2007). 17996036 10.1186/1471-2148-7-214 PMC2247476

