# Supplementary material for: Hybridogenesis and a potential case of R2 non-LTR retrotransposon horizontal transmission in Bacillus stick insects (Insecta Phasmida)
Source: Sci Rep. 2017 Feb 6;7:41946. doi: 10.1038/srep41946 (PMC5292737; doi:10.1038/srep41946)
Supplement: Supplementary Information [file srep41946-s1.pdf]

Supplementary Information

Hybridogenesis and a potential case of R2 non-LTR retrotransposon horizontal transmission in *Bacillus* stick insects (Insecta Phasmida)

Claudia Scavariello, Andrea Luchetti, Francesco Martoni, Livia Bonandin and Barbara Mantovani

Table S1. Primers used for R2 full-length elements PCR amplification and sequencing. Taxon-specificity of each primer is indicated (*Ba* = *B. atticus*; *Bgg* = *B. grandii grandii*; *Bgb* = *B. grandii benazzii*; *Bgm* = *B. grandii maretimi*).

**Primer name: Sequence 5' -> 3'**	**Reference**	**Taxon**
R2IF1:<br>AAGCARGGNGAYCCNCTNTC	22	*Ba; Bgg; Bgb; Bgm*
28S-F2:<br>GTCAAAGTGAAGAAATTCAACGAAG	46	*Ba; Bgg; Bgb; Bgm*
28SFBgg:<br>GAATCCGACTGTCTAATTAAAACAAAG	this study	*Ba; Bgg; Bgb; Bgm*
28S-R:<br>TCCATTGCTGCGCGTCACTAATTAGATGAC	32	*Ba; Bgg; Bgb; Bgm*
BggBdin3:<br>TATGATATTCAACATGGTTATTG	46	*Ba; Bgg*
BggR2Arin:<br>CGATCTAGTAGGTACTTCACACATGCA	this study	*Ba; Bgg*
Ins2_F:<br>GGACAAGCGCACAGAGTCAG	this study	*Bgg; Bgb; Bgm*
Ins2_R:<br>GGACATCCTGTTGGCGATTAC	this study	*Bgg; Bgb; Bgm*
BaadinATT:<br>ACCTCTCGCTGGCTCATTAC	this study	*Ba*
Bgg2rin:<br>AAAAAGTCCTCTAACACTTGTATCTTC	this study	*Bgg*
Bgg2din:<br>GTTAGTTTTACCCTACTGACGACCA	this study	*Bgg*
Bgg3rin:<br>ACCCCAAAACAAGGAAGGAG	this study	*Bgg*
Bgg3din:<br>TAGTATGGATTCGAGGATTAAGGAA	this study	*Bgg*
Bgg4rin:<br>GGCGACCTATTACGATTTCAAGTAT	this study	*Bgg*
Bgg4din:<br>GCACAAGTCTTTTTGGTTCATAGAT	this study	*Bgg*
Bgbrin:<br>GGTCTCTCCATTCCCTCCTATC	this study	*Bgb*
Bgbdin:	this study	*Bgb*

CCACTCCATTCAATACAGCATCT		
Bgb2rin: CACCGTCTTTCTTTGGTATTAGTGT	this study	*Bgb*
Bgb2din: AGCTCGCTCTTCCTCCTCTC	this study	*Bgb*
Bgb3din: GATCTTCACCTCCCTCATCATCAAT	this study	*Bgb*
Bgmrin: CTCCATTCAATACAGCATCTCC	this study	*Bgm*
Bgm2rin: CAGTGGGTTCGCTAGATAGTAGGT	this study	*Bgm*
Bgm2din: TTAAGGGGTCTAAGAAGGTGGAG	this study	*Bgm*
Bgm3rin: GTGTGTTTAATCGCCGTACAGTT	this study	*Bgm*
Ins_F: CTTTTGGAAGCCCTTGACCC	this study	*Bgb; Bgm*

Table S2. List of R2 sequences used for the phylogenetic analysis reported in Figure 2.

**Host species**	**Reference**
Acyrthosiphon pisum (aphid)	23
Bombus terrestris (bumblebee)	23
Bombus impatiens (bumblebee)	23
Camponotus floridanus (ant)	23
Drosophila melanogaster (fruit fly)	35
Drosophila simulans (fruit fly)	35
Harpegnathos saltator (ant)	23
Kalotermes flavicollis LI (termite)	51
Lepidururs arcticus (tadpole shrimp)	46,48
Lepidurus couesii (tadpole shrimp)	46,48
Lepidurus apus lubbockii (tadpole shrimp)	46,48
Linepithema humile (ant)	23
Limulus polyphemus (horseshoe crab)	53
Megachile rotundata (bee)	23
Nasonia vitripennis R2A (jewel wasp)	50
Nasonia vitripennis R2B (jewel wasp)	50
Nasonia giraulti R2C (jewel wasp)	50
Pogonomyrmex barbatus (ant)	23
Pogonomyrmex barbatus B (ant)	23
Popillia japonica (japanese beetle)	51
Porcellio scaber (rough woodlouse)	49
Reticulitermes balkanensis (termite)	47
Reticuitermes grassei (termite)	47
Reticulitermes lucifugus (termite)	47
Reticulitermes urbis (termite)	47
Schistosoma mansoni A (bloodfluke)	22
Schistosoma japonicum (bloodfluke)	23
Schmidtea mediterranea (freshwater planarian)	23
Solenopsis invicta (ant)	23
Tenebrio molitor A (mealworm)	52
Tenebrio molitor B (mealworm)	52
Tetranychus urticae (red spider)	23
Tribolium castaneum A (beetle)	23
Tribolium castaneum B (beetle)	23
Tribolium castaneum C (beetle)	23
Triops longicaudatus (tadpole shrimp)	22
Triops cancriformis (tadpole shrimp)	45,46

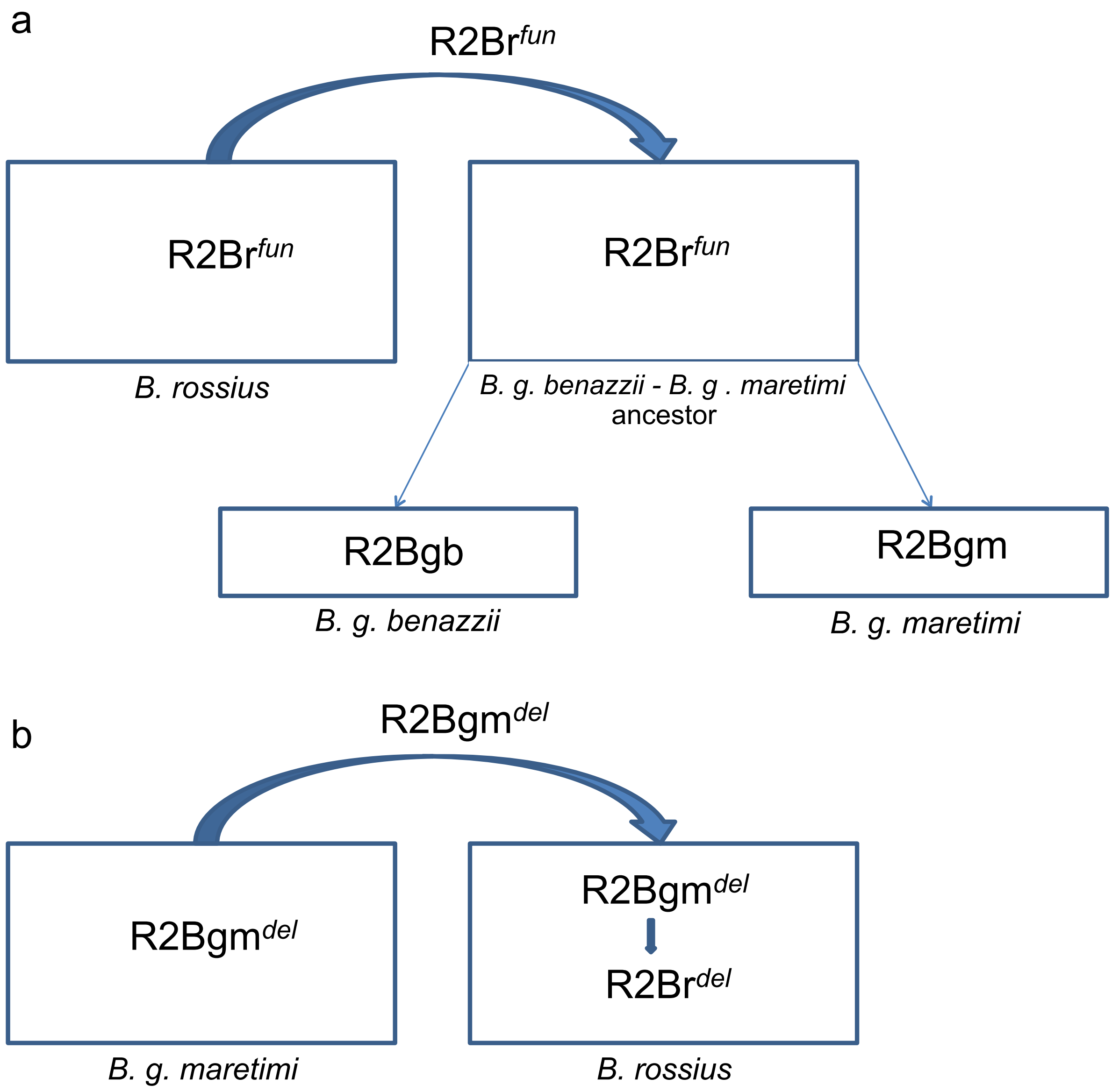

Figure S1: Summary scheme of the two possible HT events in *Bacillus* stick insects. a) The HT event transferring R2Br^{fun} element from *B. rossius* to the common ancestor of *B. g. benazzii/B. g. maretimi*. b) HT event involving the transfer of the R2Bgm^{del} element from *B. g. maretimi* to *B. rossius.*

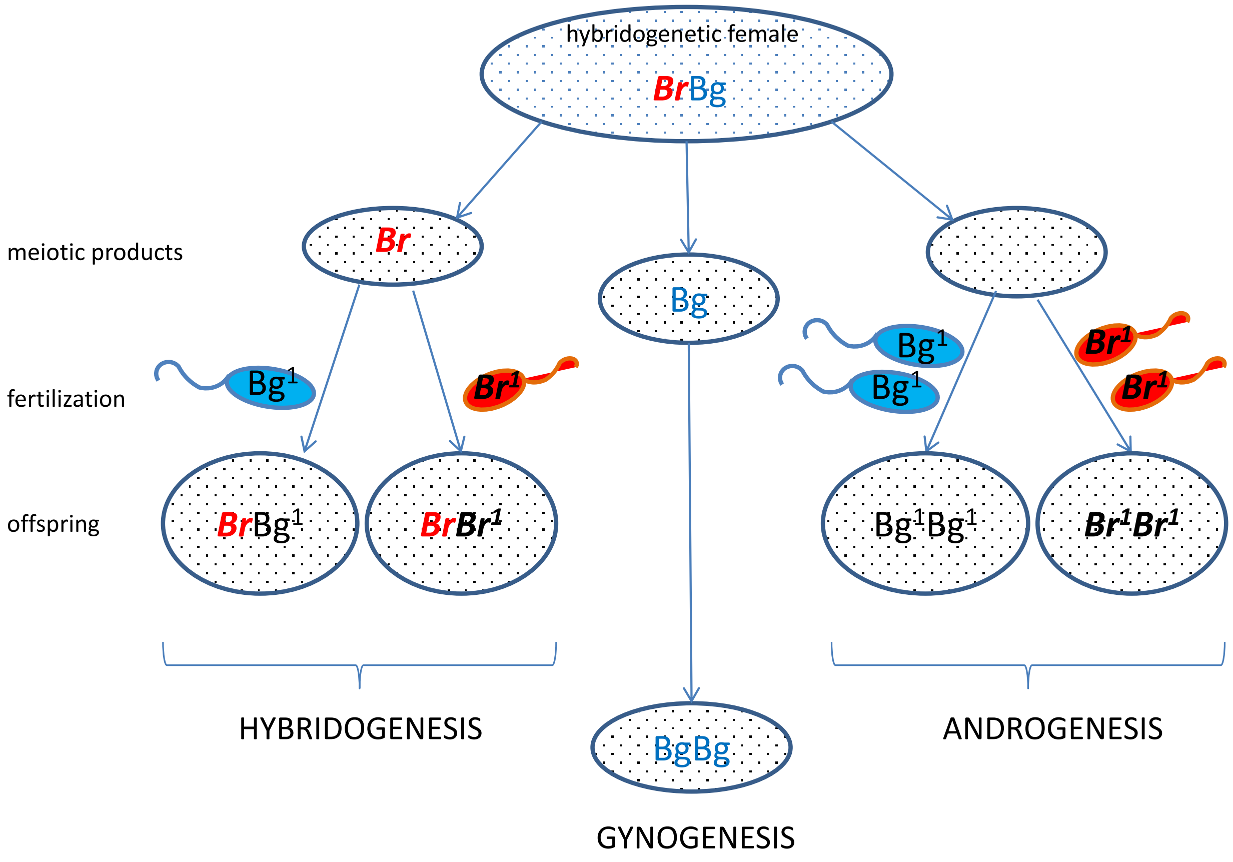

Figure S2: The hybrid lineages of *Bacillus* genus (*B. rossius*/*B. grandii*; *Br*Bg) reproduce by hybridogenesis, gynogenesis and androgenesis. Hybridogenesis takes place with the elimination of the paternal genome (Bg) and only the maternal haploid chromosome set (*Br*) is passed to the offspring in a hemiclonal way. These eggs can be fertilized by a *B. grandii* male (Bg^1), thus restoring the hybrid condition ($BrBg^1$), or by a *B. rossius* male (Br^1), resulting in a full *B. rossius* genome ($BrBr^1$). In the gynogenetic reproduction the eggs contain only the paternal haploset (Bg) that doubles at the development onset (BgBg); their activation requires sperm entrance but nuclei mixis does not take place. In androgenesis, both maternal (*Br*) and paternal (Bg) haplosets are excluded from the egg; the formation of a new diploid nucleus takes place by fusion of two other sperm pronuclei (Bg^1 or Br^1).

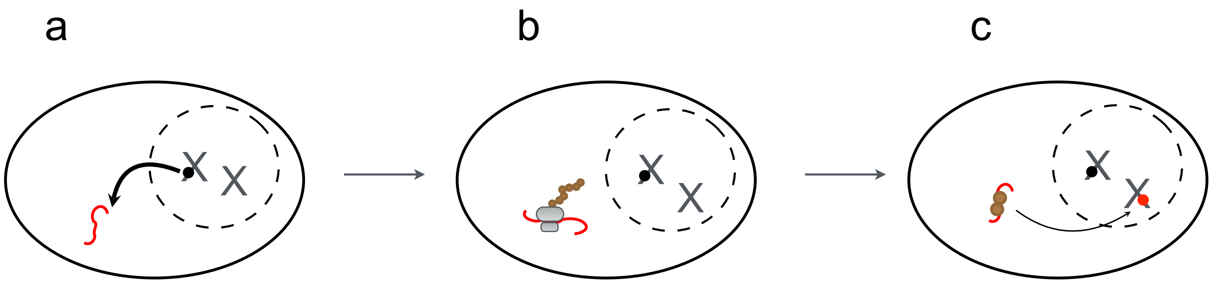

Figure S3: Mechanism of retrotransposition. a) An RNA copy of the TE is produced and exported out of the nucleus. b) In the cytoplasm the coding region is translated by the host cell machinery into a polyprotein containing a binding domain, a reverse transcriptase domain and an endonuclease domain. c) Then, the polyprotein binds the TE's RNA and the complex is reintroduced into the nucleus where the insertion reaction takes place.

Supplementary Material S1. Sequences of newly characterized R2 elements.

>R2Br^{del}
CCCTGCACTAGTCTGTGGGTTGCGGAGATAGTAGTTTAGCAAGTAGCGACCTCTGCGTGGTTCCCGCCACTAGTAGCCCT
AGTAAGGAGTAATCCTAAAGAGGTGAAGCTAACAGCTACCTCTGCGTTGGTACCCGGGGCTGGCCGCCCCGGAAGTTGTA
CCTCAATGCTAGCTAGTTCTTTCAAAAAGAAGCCTCGGATGGTGAGCTCGTCCAAAAGTGGCAGCTCGCGTAACGACGCG
CCCACTGGTGTCGTCGTGCCAGCATCGGAGGAGGGTGTTGAGTTACCTAGTCTTGACAAGAAAGTTGGATTTGGTTGTGA
ATTTCCCGGCTGTCCTAGGGTGTTCACGACCAAGACGGACGTGGGGTCCACCATCAAAAGGCCCACATGGATTGGTATG
ATGCACGACAGAAGCTTGGCGGCGTGGAAGCGCGCTGGACAAGAGAGGAGTCAGCCCTGATGGCGCGTGAGGAGGCTAAG
GCTGGGACGCAAAACGCTAACAAGACGAATCAGTTCCTGCAGTTGGTTTTGCCGGACAGGACACTGGATTCAATTAAGAG
CCACAGGAGGTCGGCCCAGTACAAGGAACTGGTCCTGCAGGCAATGGGTGCCTTGTCGGATTTGGAGAAATGTGCTGGCC
CCTCCCAAATAGCTGCTGCTGAGCTTAGTCCTCCTCTTTCCTCGGCAGTGCTGGGAACCGGTGAAACGGGTGGTGGTGGA
TCCGGTGAGCATGGGTCGGGTGATGCGCCAGGCAGTTCCCGTGTGGAACACTTGAGTGCGCTGCTAGACGGTCTGCAGCC
CGGTCCGGTTGCTGATCGGCTTCGCGTAATTGTTCGGTCGGTTGATGACTGGTCCCGGGCACGCCTGCACCAGGAGATTG
GTTGGTATCTCCGCGATCTCTTTTGGAAGCCCTTGACCCCAAACCTGGCTAGGGTGTCACTTCCATCTAAAGACAAGGTG
TCACGGCGCAGACTCAGGCGGGCGGACTATGGTCGAGTCCAGAGGGCGTGGAAGCGCTGTCGGAACACATGTGCGTCCAG
GCACATTCAGTTGGACCCTCGCCAGAAAGCTTTTCGATCTCTGGATAGTTGTTCAGAAGGGGTCTTCTTGCTGGACTTCA
TTCTTGGACATGCCAGACGTAACCACCGACCGGTGCACTTGGCGTCTCTCGACGTGGCCAAGGCATTTGACTCTGTGTCG
CACGCAGCCATCCTTGATGTACTTCGATCATTTGGTGTCCCGGATCAAATGGTTGAGTACATAGCATCGGTGTACGCAGA
CTCCAGGACACGGCTCCAGGGTGATGGGTGGCAATCCCATGCTATTCACCCCACTTGTGGGGTTAAGCAGGGTGATCCGC
TCTCACCTATGATATTCAATATGGTTATTGACCGCCTGTTCACTTTGTTCCCTCGGGACACGGGAGTGAAGGTGGGAGAT
ACTGTATTGAATGGAATGG

>R2Bgm^{del}
CCCTGCACTAGTCTGTGGGTTGCGGAGATAGTAGTTTAGCAAGTAGCGACCTCTGCGTGGTTCCCGCCACTAGTAGCCCT
AGTAAGGAGTAATCCTAAAAAGGTGAAGCTAACAGCTACCTCTGCGTTGGTACCCGGGGCTGGCCGCCCCGGAAGTTGTA
CCTCAATGCTGGCTAGTTCTTTCAAAAAGAAGCCTCGGATGGTGAGCTCGTCCAAAAGTGGCAGCTCACGTAACGACGCG
CCCACTGGTGTCGTCGTGCCAGCATCGGAGGAGGGTGTTGAGTTACCTAGTCTTGACAAGAAAGTTGGATTTGGTTGTGA
ATTTCCCGGCTGTCCTAGGGTGTTCACGACCAAGACGGACGTGGGGTCCACCATCAAAAAGCCCACATGGATTGGTATG
ATGCACGACAGAAGCTTGGCGGCGTGAAAGCGCGCTGGACAAGAGAGGAGTCAGCCCTGATGGCGCGTGAGGAGGCTAAG
GCTGGGACGCAAAACGCTAACAAGATGAATCAGTTCCTGCAGTTGGTTTTGCCGGACAGGACACTGGATTCAATTAAGAG
CCACAGGAGGTCGGCCCAGTACAAGGAACTGGTCCTGCAGGCAATGGGTGCCTTGTCGGATTTGGAGAAATGTGCTGGCC
CCTCCCAAATAGCTGCTGCTGAGCTTAGTACTCCTCTTTCCTCGGCAGTGCTGGGAACCGGTGAAACGGGTGGTGGTGGA
TCCGGTGAGCATGGGTCGGGTGATGCGCCAGGCAGTTCCCGTGTGGAACACTTGAGTGCGCTGCTAGACGGTCTGCAGCC
CGGTCCGGTTGTTGATCGGCTTCGCGTAATTGTTCGGTCGGTTGATGACTGGTCCCGGGCACGCCTGCACCAGGTGATTG
GTTGGTATCTCCGCGATCTCTTTTGGAAGCCCTTGACCCCAAACCTGGCTAGGGTGTCACTTCCATCTAAAGACAAGGTG
TCACGGCGCAGACTCAGGCGGGCGGACTATGGTCGAGTCCAGAGGGCGTGGAAGCGCTGTCGGAACACATGTGCGTCCAG
GCACATTCAGTTGGACCCTCGCCAGAAAGCTTTTCGATCTCTGGATGGTTGTTCAGAAGGGGTCTTCTTGCTGGACTTCA
TTCTTGGACATGCCAGACGTAACCACCGACCGGTGCACTTGGCGTCTCTCGACGTGGCCAAGGCATTTGACTCTGTGTCG
CACGCAGCCATCCTTGATGTACTTCGATCATTTGGTGTCCCGGATCAAATGGTTGAGTACATAGCATCGGTGTACGCAGA
CTCCAGGACACGGCTCCAGGGTGATGGGTGGCAATCCCATGCTATTCACCCCACTTGTGGGGTTAAGCAGGGGGACCCGC
TGTCACCTATGATATTCAATATGGTTATTGACCGCCTGTTCACTTTGTTCCCTCGGGACACGGGAGTGAAGGTGGGAGAT
GCTGTATTGAATGGAGTGGGATACGCAGACGACCTTGTGTTATTCACCACAACACCTGTTGGACTGCAGCAGCTGCTTGA
TATTACTGCCGAATATCTGTCGCAGTGTGGCTTGCGTGTAAACGCTGCCAAATGCTTTAGTGTATCCCTTGCAATTGTCC
CTCATGAGAGGGAAGTGGTGGTTGCAACTAAACATCGATTCAATTGTCTGGGCCAACCTATTCCGGCACTTAAGAGGTCC
GATCAATGGAAATATCTGGGAGTTCCTTTCTCTCCTGAAGGTCGGCTGAAGATTGATCCCCTCGGTCGGCTTAAGGATGA
ATTGGAAAAGCTTCGAAGGGCTCCACTGAAACCTCAGCAAAGGCTTTATGCCTTACGTACTGTGGTTGTGCCTGGCCTCT
ACCACTTGCTAGTGCTTGGAGGAACGACCATCAGTTCGTTGAATAGGCTTGACATTGCGGTTCGGTCTACGGTGCGCAAG
TGGCTGAGCCTGCCGCATGATGTGCCCAACGCGTATATCCACGCTGACGCTCGGGATGGGGGTCTCTCCATTCCCTCCTA
TCGTTGGACGGCTCCCCGGCTCCGTTTCCATAGGCTGAAGGCGCTCTCTGTCCTTTGTGACGGGGGAGGTCCGGAAGAAA
TGGTTGCGCGTGTTGGGGAGGAGATCAAGCGGGCTTCGGCTCGCTTACAAGATCATGGTATGTCCATCGATACCCGTAGT
GCCTACAGAGTTCGCTTTGCAAGGTTACTACATACCTCCAACGATGGAGCACCTCTTAAGGGGTCTAAGAAGGTGGAGGG
CCAGCATCGGTGGGTTACCGATGGGTCTCTCATGCTGTCGGGTCGTGACTACATTGCTTGCAATTGGGTCAGGATCAACT
CTATCCCGCTCCGAAAGCGGACTGCTCGCGGACGTGTCCGTGACACCCGCTGCCGGGCGGGATGTGACAGCACTGAGACA
TTACATCATGTGTTGCAGCAATGTCATCGGACTCATGACATGCGAATCAACAGGCATAATGCATGTGTGAAGTATTTACT
AGATCGCCAACGCTCTCGTGGTAAGAAGGTATTCTGGGAACCGAATTTCCACACGGCAGAAGGTCTATTGAAGCCTGACT
CTGTCATCCTTCATGACGCGTCTACTGCGGTAGTCGTTGATGCTTTGGTGGCTGGAGAGAGGTCGGATTTAGACCGAGAG
CACGACCGCAAGGTCAGCAAGTATGAACCACTGGCGGATCTAGTAAAAGACCGGTACTCTGTCGATAAAGCGATCTTCAC
CTCCCTCATCATCAATGCGCGTGGTGTGTGGGGGGGAAAATCGTTTTCGCATCTATCAAAACTGAGACTGCTTGGCATCT
CTGATGCCAAGGTCCTGTCAACCCGAGTTCTGCTCGGGGGGATGGGGGCTGCACGTGTGTTTAATCGCCGTACAGTTGTG
CGTGGGAGGGTCAATGGCTGGTGACTCTCAACCTCCTTTAATTATGTGCTACCTCCTCCAGCTTGAGGTATAATATAGAG
GGTTTTTTTAGTGGGTAGTCACCTTGTATTGGGTGGAGTCTCACGCGAAATGCCACTGGTCACCTGGCATGAGTGCGTAA
TGCATTTTTCCCGCTCT

>R2Bgm
CCCTGCACTAGTCTGTGGGTTGCGGAGATAGTAGTTTAGCAAGTAGCGACCTCTGCGTGGTTCCCGCCACTAGTAGCCTT
AGTAAGGAGTAATCCTAAAAAGGTGAAGCTAACAGCTACCTCTGCGTTGGTACCCGGGGCTGGCCGCCCCGGAAGTTGTA
CCTCAATGCTGGCTAGTTCTTTCAAAAAGAAGCCTCGGATGGTGAGCTCGTCCAAAAGTGGCAGCTCGCGTAACGACGCG
CCCACTGGTGTCGTCGTGCCAGCATCGGAGGAGGGTGTTGAGTTACCTAGTCTTGACAAGAAAGTTGGATTTGGTTGTGA
ATTTCCCGGCTGTCCTAGGGTGTTCACGACCAAGACGGGACGTGGGGTCCACCATCAAAAAGCCCACATGGATTGGTATG
ATGCACGACAGAAGCTTGGCGGCGTGAAAGCGCGCTGGACAAGAGAGGAGTCAGCCCTGATGGCGCGTGAGGAGGCTAAG
GCTGGGACGCAAAACGCTAACAAGATGAATCAGTTCCTGCAGTTGGTTTTGCCGGACAGGACACTGGATTCAATTAAGAG
CCACAGGAGGTCGGCCCAGTACAAGGAACTGGTCCTGCAGGCAATGGGTGCCTTGTCGGATTTGGAGAAATGTGCTGGCC
CCTCCCAAATAGCTGCTGCTGAGCTTAGTACTCCTCTTTCCTCGGCAGTGCTGGGAACCGGTGAAACGGGTGGTGGTGGA
TCCGGTGAGCATGGGTCGGGTGATGCGCCAGGCAGTTCCCGTGTGGAACACTTGAGTGCGCTGCTAGACGGTCTGCAGCC
CGGTCCGGTTGTTGATCGGCTTCGCGTAATTGTTCGGTCGGTTGATGACTGGTCCCGGGCACGCCTGCACTAGGAGATTG
GTTGGTATCTCCGCGATCTCTTTTGGAAGCCCTTGACCCCAAACCTGGCTAGGGTGTCACTTCCATCTAAAGACAAGGTG
TCACGGCGCAGACTCAGGCGGGCGGACTATGGTCGAGTCCAGAGGGCGTGGAAGCGCTGTCGGAACACATGCTTACGCGA
TCTCTTGAGGGACAAGCGCACAGAGTCAGCACCACCCAAAGATCTAATGGTGCCTTATTGGGAGAGTGTGCTGCGTTCGG
GATCGTCCTGTACTCCCGGCCAAAGAGGTCGGACAGCCGAGATAACAGAGTTGTGGGACCCGGTGTCCTCGAGAGAGGTT
GAGCTCGCACTTCCTCCTCTCGGGACAGCACCGGGTCCAGATTCCTTTACACCAAAGGACTTCAGGGCGGTGCCGTCTGT
TGTTTGGGCCTGCATTTATAACATCTTTATGCTGTGTGGCCGGCTCCCGGATCATCTCCTCGAGTCGCGCACTACACTGA
TACCAAAGAAAGACGGTGCTTGTAACCCTGAAGATTTCCGGCCGATAACCGTCTCCTCTGTTGTGGTTAGATGCTTTCAT
AAGGTGATCGCCAACAGGATGTCCAGGCACATTCAGTTGGACCCTCGCCAGAAAGCTTTTCGATCTCTGGATGGTTGTTC
AGAAGGGGTCTTCTTGCTGGACTTCATTCTTGGACATGCCAGACGTAACCACCGACCGGTGCACTTGGCGTCTCTCGACG
TGGCCAAGGCATTTGACTCTGTGTCGCACGCAGCCATCCTTGATGTACTTCGATCATTTGGTGTCCCGGATCAAATGGTT
GAGTACATAGCATCGGTGTACGCAGACTCCAGGACACGGCTCCAGGGTGATGGGTGGCAATCCCATGCTATTCACCCCAC
TTGTGGGGTTAAGCAGGGTGATCCGCTCTCACCTATGATATTCAATATGGTTATTGACCGCCTGTTCACTTTGTTCCCTC
GGGACACGGGAGTGAAGGTGGGAGATACTGTATTGAATGGAATGGGATACGCAGACGACCTTGTGTTATTCACCACAACA
CCTGTTGGACTGCAGCAGCTGCTTGATATTACTGCCGAATATCTGTCGCAGTGTGGCTTGCGTGTAAACGCTGCCAAATG
CTTTAGTGTATCCCTTGCAATTGTCCCTCATGAGAGGGAAGTGGTGGTTGCAACTAAACATCGATTCAATTGTCTGGGCC
AACCTATTCCGGCACTTAAGAGGTCCGATCAATGGAAATATCTGGGAGTTCCTTTCTCTCCTGAAGGTCGGCTGAAGATT
GATCCCCTCGGTCGGCTTAAGGATGAATTGGAAAAGCTTCGAAGGGCTCCACTGAAACCTCAGCAAAGGCTTTATGCCTT
ACGTACTGTGGTTGTGCCTGGCCTCTACCACTTGCTAGTGCTTGGAGGAACGACCATCAGTTCGTTGAATAGGCTTGACA
TTGCGGTTCGGTCTACGGTGCGCAAGTGGCTGAGCCTGCCGCATGATGTGCCCAACGCGTATATCCACGCTGACGCTCGG
GATGGGGGTCTCTCCATTCCCTCCTATCGTTGGACGGCTCCCCGGCTCCGTTTCCATAGGCTGAAGGCGCTCTCTGTCCT
TTGTGACGGGGGAGGTCCGGAAGAAATGGTTGCGCGTGTTGGGGAGGAGATCAAGCGGGCTTCGGCTCGCTTACAAGATC
ATGGTATGTCCATCGATACCCGTAGTGCCTACAGAGTTCGCTTTGCAAGGTTACTACATACCTCCAACGATGGAGCACCT
CTTAAGGGGTCTAAGAAGGTGGAGGGCCAGCATCGGTGGGTTACCGATGGGTCTCTCATGCTGTCGGGTCGTGACTACAT
TGCTTGCAATTGGGTCAGGATCAACTCTATCCCGCTCCGAAAGCGGACTGCTCGCGGACGTGTCCGTGACACCCGCTGCC
GGGCGGGATGTGACAGCACTGAGACATTACATCATGTGTTGCAGCAATGTCATCGGACTCATGACATGCGAATCAACAGG
CATAATGCATGTGTGAAGTATTTACTAGATCGCCAACGCTCTCGTGGTAAGAAGGTATTCTGGGAACCGAATTTCCACAC
GGCAGAAGGTCTATTGAAGCCTGACTCTGTCATCCTTCATGACGCGTCTACTGCGGTAGTCGTTGATGCTTTGGTGGCTG
GAGAGAGGTCGGATTTAGACCGAGAGCACGACCGCAAGGTCAGCAAGTATGAACCACTGGCGGATCTAGTAAAAGACCGG
TACTCTGTCGATAAAGCGATCTTCACCTCCCTCATCATCAATGCGCGTGGTGTGTGGGGGGGAAAATCGTTTTCGCATCT
ATCAAAACTGAGACTGCTTGGCATCTCTGATGCCAAGGTCCTGTCAACCCGAGTTCTGCTCGGGGGGATGGGGGCTGCAC
GTGTGTTTAATCGCCGTACAGTTGTGCGTGGGAGGGTCAATGGCTGGTGACTCTCAACCTCCTTTAATTATGTGCTACCT
CCTCCAGCTTGAGGTATAATATAGAGGGTTTTTTAGTGGGTAGTCACCTTGTATTGGGTGGAGTCTCACGCGAAATGCC
ACTGGTCACCTGGCATGAGTGCGTAATGCATTTTTCCCGCTCT

>R2Bgb
CCCTCCACTAGTCTGGAGGTTGCGGAGATAGTAGTTAGCAAGTAGCGACCTCTGCGTGGTTCCCGCCACTAGTGGCCGTA
GTAGGGAGTAATCCTGAAAAGGTGAAGCTAACAGCTACCTCTGCGCTGGTACCCGGGGCTGGCCGCCCCGGAAGTGTACC
TCATGCTGGCCAGTTCTTTCAAAAAGAAGCCTCGGATGGTGAGCTCGCCCAAAAGTGGCAGCTCATGTAACGACGCGCCC
ACTGGTGTCGTCGTGCCAGCATCGAAGGAGAGTGTTGAGTTACCTAGTCTTGACAAAGAAGTTGGATATGGTTGTGAATT
TCCCGGCTGTCCTAGGGTATTCACAACCAAGACGGGACGTGGGGTCCACCATCGAAAAGCCCACGAGGATTGGTATGATG
CGCGACAGAAGCTTGATTACGGGAAAGCGCGCTGGACAAGAGAGGAGTCAGCCCTGATGGCGCGTGAGGAGGCTAAGGCT
AGGATGCAAAATGCTAACAATATGGACCAGGTCCTGCAGTTGGTTTTGCCGGACAGGACACTGGATTCAATAATGAGCCA
CAGGAGATCGGCACAGTACAAGGAACTGGTCCTGCAGGCAATGGGTGCCTTGTCGAATTCAGGGAAATGTGCTGGCCCCT
CCCAATTAGCAAATGCTGAGCTTAGTACTCCTCTTTCTTCGGCAGTGCTGGGAACCGGTGAACCGGGTGGTGGGGGATCC
GGTGAGCATGGGTCGGGTGATGCGCCAGGCAGTTCCCGTGTGGAACACTTGAGTGCGCTGCTAGACGGTCTGCAGCCCGG
TCCGGTTGTTGATCGGCTTCGCGTAATTGTTCGGTCGGTTGACGACTGGTCCCGGGCACGCCTGCACCAGGAGATTGGTT
GGTATCTCCGCGATCTCTTTTGGAAGCCCTTGACCCCAAACCTGGCTAGGGTGTCACTTCCATCTAAAGACAAGGTGTCA
CGGCGCAGACTCAGGCGGGCTGACTATGGTCGAGTCCAGAGGGCGTGGAAGCGCTGTCGGAACACATGCTTACGCGATCT
CTTGAGGGACAAGCGCACAGAGTCAGCACCACCCAAAGATCTAATGGTGCCTTATTGGGAGAGTGTGCTGCGTTCGGGAT
CGTCCTGTACTCCCGGCCAAAGAGGTCGGACAGCCGAGATAACAGAGTTGTGGGACCCGGTGTCCTCGAGAGAGGTTGAG
CTCGCTCTTCCTCCTCTCGGGACAGCACCGGGTCCAGATTCCTTTACACCAAAGGACTTCAGGGCGGTGCCGTCTGTTGT
TTGGGCCTGCATTTATAACATCTTTATGCTGTGTGGCCGGCTCCCGGATCATCTCCTCGAGTCGCGCACTACACTAATAC
CAAAGAAAGACGGTGCTTGTAACCCTGAAGATTTCCGGCCGATAACCGTCTCCTCTGTTATGGTTAGATGCTTTCATAAG
GTGATCGCCAACAGGATGTCCAGGCACATTCAGTTGGACCCTCGCCAGAAAGCTTTTCGATCTCTGGATGGTTGTTCAGA

AGGGGTCTTCTTGCTGGACTTCATTCTTGGACATGCCAGACGTAACCACCGACCGGTGCACTTGGCGTCTCTCGACGTGG
CCAAGGCATTTGACTCTGTGTCGCACGCAGCCATCCTTGATGTACTTCGATCATTTGGTGTCCCGGATCAAATGGTTGAG
TACATAGCATCGGTGTACGCAGACTCCAGGACACGGCTCCAGGGTGATGGGTGGCAATCCCATGCTATTCACCCCACTTG
TGGGGTTAAGCAGGGGGATCCGCTCTCACCCATGATATTCAATATGGTTATTGACCGCCTGTTCACTTTGTTCCCTCGGG
ACACGGGAGTGAAGGTGGGAGTTGCTGTATTGAATGGAGTGGGATACGCAGACGACCTTGTGTTATTCGCCACAACACCT
GTTGGACTGCAGCAGCTGCTTGATATTACTGCCGAATATCTGTCGCAGTGTGGCTTGCGTGTAAACGCTGCCAAATGCTT
TAGTGTATCCCTTGCAATTGTCCCTCATGAGAGGAAAGTGGTGGTTGCAACTAAACATCGATTCAATTGTCTGGGCCAAC
CTATTCCGGCACTTAAGAGGTCCGATCAATGGAAATATCTGGGAGTTCCTTTCTCTCCTGAAGGTCGGCTGAAGATTGAT
CCCCTCGGTCGGCTTAAGGATGAATTGGAAAAGCTTCGAAGGGCTCCACTGAAACCTCAGCAAAGGCTTTATGCCTTACG
TACTGTGGTTGTGCCTGGCCTCTACCACTTGCTAGTGCTTGGAGGAACGACCATCAGTTCGTTGAATAGGCTTGACATTG
CGGTTCGGTCTACGGTGCGCAAGTGGCTGAGCCTGCCGCATGATGTGCCCAACGCGTATATCCACGCTGACGCTCGGGAT
GGGGGTCTCTCCATTCCCTCCTATCGTTGGACGGCTCCCCGGCTCCGTTTCCATAGGCTGAAGGCGCTCTCTGTCCTTTG
TGACGGGGGAGGTCCGGAAGAAATGGTTGCGCGTGTTGGGGAGGAGATCAAGCGGGCTTCGGCTCGCTTACAAGATCATG
GTATGTCCATCGATACCCGTAGTGCCTACAGAGTTCGCTTTGCAAGGTTACTACATACCTCCAACGATGGAGCACCTCTT
AAGGGGTCTAAGAAGGTGGAGGGCCAGCATCAGTGGGTTACCGATGGGTCTCTCATGCTGTCGGGTCGTGACTACATTGC
TTGCAATTGGGTCAGGATCAACTCTATCCCGCTCCGAAAGCGGACTGCTCGCGGACGTGTCCGTGACACCCGCTGCCGGG
CGGGATGTGACAGCACTGAGACATTACATCATGTGTTGCAGCAATGTCATCGGACTCATGACATGCGAATCAACAGGCAT
AATGCATGTGTGAAGTACTTACTAGATCGCCAACGCTCTCGTGGTAAGAAGGTATTCTGGGAACCGAACTTCCACACGGC
AGAAGGTCTATTGAAGCCTGACTCTGTCATCCTTCATGACGCGTCTACTGCGGTAGTCGTTGATGCTTTGGTGGCTGGAG
AGAGGTCGGATTTAGACCGAGAGCACGACCGTAAGGTCAGCAAGTATGAACCACTGGCGGATCTAGTAAAAGACCGGTAC
TCTGTCGATAAAGTGATCTTCACCTCCCTCATCATCAATGCGCGTGGTGTGTGGGGGGGAAAATCGTTTTCGCATCTATC
AAAACTGAGACTGCTTGGCATCTCTGATGCCAAGGTCCTGTCAACCCGAGTTCTGCTCGGGGGGATGGGGGCTGTACGTG
TGTTTAATCGCCGTACAGTTGTGCGTGGGAGGGTCAATGGCTGGTGACTCTCAACCTCCTTTAATTATGTGCTACCTCCT
CCAGCTTGAGGTATAATATAGAGGGTTTTTTAGTGGGTAGTCACCTTGTATTGGGTGGAGTCCCACACGAAATGCCACTG
GTCACCTGGCATGAGTGCGTAATGCATTTTTCCCGCTCT

>R2Bgg^{A}

GYGGGTAAACGGCGGGAGTAACTATGACTCACCAGGTCGTAGTTAGCAAGTAGCGGCTTCTGCGTGGCTCCCGCCACTAG
TAACCCTATTAGGGAGCAATCCCGAACGGGTGAAGCCAACAGCTACCTCTGCGCTGGTACCCGGGGCTGGCCGCCCCGGA
AGTGTACCTCTCGCTGGCTCAGTATAAAAGAAATAAGCCTCGGATGGTGGGTTCGTCCAATAGTGGCAACCCCCGAAACG
ACACGCCCACTGGTGATGTCGTGCCAGCGGCTCAGGAGAGTGTAGGATCATCCAGGCAATGCCAAAGTTATGGATTCTGT
TGTGAATTTCCTGACTGCGGTCGGGTATTCTCAACCAAGTCGGGACGTGGGCTCCACCATCGTAACGCCCACCCAGATTG
GTATGACTCCCGGCAGAAGCTTGGATCCGCGAAAGTGCGCTGGACAAGTGAGGAGTCCACCCTGATGGCACGGGAAGAGG
CCAAAGTTGCGACACTCAAATCACAGCATATACATCAGGCCCTGTTGTTGGCTCTGCCGCACAGGACCCTCGATGCAATC
AAAGGCCAGAGGAGGACGGCCCAGTACAAGGAACTGGTGCGGCAGGCAGTGGATGCTCTGTCGGATTCAGGGAGATGTGC
TTGCCCCTCCCAAGCTGAGAGTGTCGAACCTAAACCTCCAACCCCCCCGGTAGCGCTGGGACCCGGTGACTTGAGTGATC
AGGAATCCGGTGAGCGTGGGCTGGGGGATCAGCCTGGTAGCTCCCAAGTGCTGGGTAGTGGGGTTCCTAGCAAGTCCCCC
GCTGCCTGCATAAGGGAGCATTTAAGTGTGCTGCCGGACAGTCTGCAGCTCGGGCCAGTCGAACGCCGTCTGAACCAGGT
GATTCGGTCGATAGACGATTGGTCTCGAGAACGCCTGCACCAGGAGATAACACGGTATCTCCGAGAGTTCTTTTGGAAAC
CCCCGACACGAGACCTGGCTAGGGGGCCAATTCCATCTCAAGACAAGGTGTCTCGGCGCAGACTTAGGCGGRCGGACTAT
GGACGAGTCCAGAGGGCATGGAAGCGGAATCGGAACACATGCTTGCGTGATCTCATGAGGGACAAGCGCACAGAGTCAGC
ACCACCCGAAGAACTTATGGTGCCTTATTGGGAGACTGTGTTTCGATCTGGAGCGTCTTGTACACCTGGTTTAAGAGGTC
GGACGGTCGACAAAACTGAGCTCTTTGAGCTATGGGAACCGGTGACTGTCAGAGAGGTTGAGCGTGCTCTTCCTCCTCTG
GGGTCAGCGCCGGGCCCAGACTCCCTGATGCCTGGGGACTTCAGGAAGGTATCGTCCAATGTTTGGGCTAGCATATTTAA
TATCTTTATGCTGTGTGGTCGGCTCCCGGATTACCTCCTCGAGTCCCGTACCACACTCATTCCCAAGAAGGACGGTGCTA
GCACTCCTGAAGAGTTTAGACCGATTACCGTTTCCTCAGTGGTGGTCCGATGCTTTCACAAGGTGATGGCCAACAGGATG
TCTAAAAGCATTCAGTTGGACCCTCGCCAGAAAGCCTTTCGATCTGTGGATGGATGCTCTGAGGGCGTTTTCCTTCTGGA
CTTCGTTCTTGGGCATGCCAGACGCAACCACCGGCCAGTTTGTCTTGCGTCTCTGGACGTTGCCAAAGCATTTGACTCTG
TGTCGCACGCAGCCATCTTGATGTGCTTCGATCATTTGGGGTACCTGGCCCAATGGTTGAGTACATAGCATCTGTGTATG
CAGGCTCTAGGACAAGGCTCCAAGGCGTTGGGTGGAGATCTCACGCTATCCACCCCACTTGTGGGGTTAAGCAGGGTGAC
CCGCTCTCGCCTATGATMTTCAACATGGTTATTGACCGCCTGTTCTCTTTGTTTCCACAAGATACAGGAGTCCGGGTGGG
AGATGCTGTGTTGAATGGAATGGGGTATGCAGACGACCTAGTGTTGTTTGCCTCGACACCTGTCGGACTGCAACAGCTGC
TGGATATTACTGCCGAATATCTCACGCAGTGTGGTCTGCGAGTGAATGCTGGGAAATGCTTCACGGTATCCCTTGCGACT
GTCCCTCATGAGAAGAAAGTGGTGGTCAATGTTAAAGATCGATTCAAGTGTCTGGGTCAACCTATTCCGGAACTAAAGAG
GACTGATCAATGGAAATATCTTGGAGTTCCCTTTTCTCCTGACGGTCGGCTTAAGGTGGACCCACTTGGCCGTCTGAAGG
ATGAACTGGAAAAACTTAGAAGGGCTCCATTGAAACCTCAGCAGAGGCTTTATGCCTTGCGGACAATGGTTATACCAAGC
CTCTATCATATGCTGGTGCTAGGAGGAACGACCATCAGTTCGTTGAATTGGCTTGACATTGTGGTTCGGGCCACCGTGCG
TAAGTGGCTGGCCTTGCCGCATGATGTACCCAACGCATACATCCATGCTGACGCTTGGGATGGGGGTCTCTCCATTCCCT
CTTATCWTTGGAGCGTTCCCCTGCAGCGCTTCCATAGGCTGAAGGCGCTTTCCCTGCTCTGCAGTGAGACTGGGCYGGAG
GACRTGGTTGCGTCTGTGGGGGACGAGATCACACRGGCAGCCGCYCGTCTGAATGATCATGGTACGACCRTCAGTACCCG
TAATGCCTACAGAGYTCGCTTTGCTAGGTTATTACATACCTCCAAYGATGGAGCACCTCTTAARGGGTCGAAGMAGGTGG
AGGGCCAGCACCGMTGGATTACCGATGGGTCKCTYATGTTRTCTGGTCGTGATTTTATTGCWTGCAATAGAATCAGGAYC
AAYGCCATTCCGCTSAGGAAGYGATGTGCWAGARGACGAGWCCATGACACTCGTTGCCGTGCGGGATGCGACAACATGGA
GACATTACATCATGTGCTGCAGCAATGTCATAGGACCCATGATGCAAGAATCAACAGACACRATGCATGTGTGAAGTACC
TACTAGATCGGCAACGCTCTCGCGGCAAAAATGTTGACAGGGAACCACATTTCCGCACGGCAGATGGTCTTTTGAAGCCT

GACTCAGTCATCCTTTATGACGCGTCTACTACGGTAGTCGTTGATGCTTTGGTGGCTGGTGAGAGGGCTGATCTAGACCG
AGAGCATGACCGTAAGGTCCAGAAGTATGCACCATTAGCCGATCTAGTTAAGGCACGGTACTCTGTCGAAAAGGTGATCT
TCTCTTCCCTCACAATCAATGCATGGGGGGTGTGGGGAGGAAGATCATTGAGGAGTCTATCAAAGCTAAGATTGCTTAGT
GTCTCTGATGCTAAGGTCCTGTCAACTCGAGTTCTGCTCGGGGGGCAGGGGCTGTACGTGTGTTTCATCGCCGTACAGGA
ATCGGTGGGAGAGCTTATGTCTGGTGATTCACACTCTCCTTATTGCAAGTACTACCTCCTTCCAGCTTGAGGTACAATAT
AGAGGGTTTTTAGTGGGTAGTCACCTTGTATCGGGGGAGTCCCAAACAACCTGCCACTGGTCACCTGGCAGGAGTGCGT
AATGTGTTTTTCCCGCTCT

>R2Bgg[B]

AGAGAAGGAAGGCAGTCGTTCGAGGTGTCGGAGCAAGCTCTAGTTATCCGAGACTAGCCTCCAACATAGTTACGGAAAGG
TCTCAAGAAGCGTCTCATATAGCCTCTCAGAGACAAGGCGACCCCCACCTCCTTGTGGTACCGCTGGTTTTCTAACGGGG
GCTACGCCGAAAGGGTGGGGACTTGTTCCCAGTGGTCTGTACAGTTGGGCACTGTGCAGACCCAGAAAAGGGCTGGGGGT
CAGGTTGGGATTTAGAGCCACGCTCTCCTGTCGCTGGAAACCCAGTCTAGCCTRCCCCCTTACACATCCAGTTCTACAGG
TGTGTTGCCACCCGCCCTGCAACTGATCATCTGTGTGGGGAAAGGGGGAGRTGATTTSCGGCAGGAATGCAGGCATGATG
AGGGTTAACTGCCCCATCATGTGGCCCTAAATAAGTTGCAGAACCAAATAAATTGCCTCGGATCGKGGTGGTCCCGCAGA
AGGGCACCCCAAAACAAGGAAGGAGGGCAAGGGGTGTTTCACCTCGAACCCGAGAAGCGCAGGAGATGCAGAGCGGCATC
GAGAGGGAGCCTGACATGTGTTCGTCTTGTCCTACACGCGAGCAAGACTTAACCGATCGGGACTTAGCTAACCGGAAGAG
ACATATTAACAGGTGCATCCAGCCACCTGCTGATGCAGATGGCTTATACCACTGTAATATAAATGGGTGCACCAAATCCT
TCCCCACCTTCTTGAGTATTCGATTGCACCAGAAGAGTGCACATCCCTCAGAATATAACAAAATGGATGAACAGTTAGCT
CAAGGAAGGAAAACTGTGAGATATACAGAAGATGTATATGAATTAATGGCAAATATTGAGCTGGATGCTCCTCCTGGTGA
TAAACAAATGAATATTAACCAGAGGATGGCTACCCTATTGAATAGAACGCCTGCGGGTATTAAGAAGGCGAGAACAAGGG
AAGGATACATGGAAGTGTTACAAAGAGTTAAGGAAGAACGTCTGGCCGCAATTATTGAAGCCCAGGCCAAGACAGAGGAT
GAACCTGGTACTATCCAAGACTTCATCAAGAATGATGAGGAGAAAATACAGGAGATGTGGAATAAAGTCGGAGATCGGGG
ATTGACGCCGTCTCTACCACTACAACTTGTAGTGAACGAGTGTGCTAAACAACCTAGAGATATGATTCTTATTGACCATA
TCATTAGCACATATTTCGCTTATGTATTATATAAAAGCGAAGTTCCTGTGGAGACGGCGAAGAAATCTAGATCCAGGCGG
AAAACCTGGAGAACTAAGAAAAGCAATTATGGCTCTTCCAAAAGAAGGGAGAGGGAGAAGGAAAGAAGTGCGAATTACGC
AAGAATCCAAGAAATGTACTGGGTTCAACCAGCCAAAGCGGCCTCTATAAATTTGGATGGAGAAAAGGACAGTGAAACCT
CTCCTCCAATGCAAGAATTTATTGAAGCATATGAACAACTATTTGCGGCCCCACCACAACCCCCTAGAATAGAGGGTAAG
CATGAGGTGCAACATGATATAAGCTCTATTATTGAACTTGAAGAGATTAATAGAGCTTTGACCCAATTGAAAGACACTAC
TCCTGGACTGGATGGAGTGGGGAAGGAGCACCTCCGTGTAATGAATAAGAATGATTTGTTCATTCTATTAAACATAATTT
GGATATTCCAAATTTTCCCATCTTACATAAGGGAAAATAGAACGGTATTGATACCGAAAAATGAAAGAGTTGAAAAGGTT
GATAATTGGCGACCTATTACGATTTCAAGTATTAATATGAGAATCCTGAATAGGATCATTAATTGGCGCCTAGAAGCAAT
TCCTGAATTGCATGCTAGCCAGAAGGGATATACACGGGAGGGTGGCATCCTTTGTAATTCCTTAATCCTCGAATCCATAC
TAAATCGAATTCCCGCGGCCGCCATGGCGGCCGGGGGAATTCGATTACCCCAAAACAAGGAAGGAGGGCAAGGGGTGTTT
CACCTCGAACCCGAGAAGCGCAGCCTGTACAGCACGATTTGAGCGGAATTATACTCATGGAAGAGGTGGAGAGAGCATTA
TATAGTATGGCGGACTCTTCTCCTGGCTTGGATGGCCTTAGAAAAGAACACCTACAATCTATGAACCAAAAAGACTTGTG
CATATTGACAAACATAATGTGGATCCTACATTATACTCCTTCCATTATGAGGAGCAATCGCACAGTATTAATCCCCAAGG
GGAACAGCGACTTGTTGCAAGAAGGCAACCGGAGGCCAGTCACTATATCCAGTGTCCTTATAAGACTGGTTAGTAAGATT
ATATATTTCAGAATGGAGGCTGTTATCTGCCACCATCAAAGTCAGAAAGGATTCACAAGAGAGGATGGAATATTAATTAA
TTCCTTAATCCTCGAATCCATACTAAAATTACATAAGAACAAGGCAATGCCAATTATTGCTATTAGCATTGACCTACGCA
AAGCTTTCGATAGCGTTTGCTTVGAATCCATTCTGAACGCCCTATATGCTAATGGCATCGATGAGCACACTATTAGTCAT
ATTATGACGATGTACACGAACAATGTGACTGTTCTACAATGCAAAGGCGAGAGCTCTGAAGACCTTAGAGTGAATAGAGG
CGTCAGACAAGGGGATCCCCTTAGTGGGATCTTGTTCAACCTTGTACTTGACCCTCTATTAGATGCTTTGGAGAAGGAAG
GGAAAGGGATCCCCCTTCAAAATGCAAATGTACACTTGCTTTAGCGTTTGCRGATGATGTCGTTATGCTTGCTTCAGAGT
GCATACGAAATGAAAAAGAAGATACAAGTGTTAGAGGACTTTTTAAAGGTAACCTGCATGCAGTGCAATACAAAGAAATG
TTTTTCATACCAGCTTGTACATATTCCCAATACCAAGAGGGTATTTGTACAGACTGAGAGTCTTCTGCAAATTTGTGGGA
ACGATATCAAAGCCCTGAATACGGTGTCCCAGTGGAAATATCTGGGACAGGTGTACACAAACGCTGCATCGCAGGCCCTT
GGACCGGACTTACTTAACAACAAGCTGTTGAAATTGATGACTGCCCCGTTAAAGCCGTGGCAAAAGCTGACTATGCTCAA
TAGGTACATTATACCTAGAACATACCATCGCCTAATGTGCATGGATATTACAGCGAAGAGATTGAAGGCGTATGATAAAA
CTATTGTTACCTTTGTCAAAAAGGTACTCCATCTGCCTATTACAATGCCTACAGCTTTCCTCTACACTCCTATACGAAGT
GGAGGATTGTCGATTCCTTGCCTCCGGCACCAAATCGGGGCAGTGTATACTAGGAGACTAAACAAATTGGCAAGGTCCAC
CGACCCACGCGTAATTGCAGTAATGCAGAGTGCCCCTGTGCAACAATTACGCAACAAACTAAGTGTCATCCTGGGTCCGG
TGGGACCTTCGAAAGAGGCCAGCAAGAAATTCTGGACCTCTATACTATATAACGAGACTTCATTAGGCAGTGGCCTGAAG
CATATGGTACCCCAAGGGGGCAAGTGGATATTGAACCCTCCCTCATGGTGGTCGGGAAGGGAATATATTGCCGCGGTCCA
TCTGCGACTGGGGCTACTGCCAACTATAGGCCCACCCTATGTTCCAGAGGAGCAGGCCAAATGTCGTAGCAACATTTGCC
ATGTGAGAGAATCTTTATCACATGTTATTCAGCGGTGTCCGATAACACACTACCAGAGGATTGAGAGACATGACAAGGCT
AACGGCCTTTTGTACGCTGCTATTGCTGCKAAMRAGWYTYCAGTCTTTTTGGAACCCAGGCTACATGCCTCTTTAGGCAG
TGGCCTGAAGCATATGGTACCCCAAGGGGGCAAGTGGATATTGAACCCTCCCTCATGGTGGTCGGGAAGGGAATATATTG
CCGCGGTCCATCTGCGACTGGGGCTACTGCCAACTATAGGCCCACCCTATGTTCCAGAGGAGCAGGCCAAATGTCGTAGC
AACATTTGCCATGTGAGAGAATCTTTATCACATGTTATTCAGCGGTGTCCGATAACACACTACCAGAGGATTGAGAGACA
TGACAAGGCTAACGGCCTTTTGTACGCTGCTATTGCTGCGAAAAAGTTTCCAGTCTTTTTGGAACCCAGGCTACATGCCT
CTGGTGCGCGCTATATGCCCGACATTATTGTCGTTGATGGCGACAAAGCCATAATCGTGGATACTACAATTTGTTGGGAA
TATAAAGACTCCATGCAAGATGCTTATATGATTAAGAAGACCAAATACGACATCCCTGCCGTAAACGAGAAAGTTCAAAC
AAAATATGGTGTTAATGAGGTACACCATATGCCTTTTGTGATTGGGGCTCGAGGCTCTTGGTTTAGTGAGAACAAGGCCA
TCTGGAAACGATTTAAATTGCCTGCTTCCCTAATTGGCATTATTATACATAATGTCCTGTTGTTTGGGGTAAAGATACAC

TATTCATTTATGGCATGTATATGGAGAAAGGCCCGTTACACAAGGAGTGCCTCCCAATGCCGAAGAGGAGAGAGAAGATA
TCCCCAGTCCTGAAGATCATGGAAGAAGAAAGGAAGATAGCCGAGATGTTTCACTGCTGCATCGAGCTCCAGTATGAAGA
AAGAAGAGAGTACGTCCACGACAAGTCCGGAAGACGACCCTGTGAAGAGGAGAAGAGGGCCCAGCTGCTCCTCTTCCCCC
CTGGATATTGAGTGCTTCCTGTTACTGCCTTTGTATCTTTGTGCATCTGTATTTGTATTTGTAACTATGTTACAGCACAT
ATTTATGTAATAAATGAGTTCTAGCAAAA

>R2Ba
AGGTCGCAGTTAGCAAGTAGCGGCCTCTGCGTGGCTCCCGCCACTAGTAGCCCTATTAGGGAGTAATCCCGAACGGGTGA
AGCTAACAGCTACCTCTGCGATGGTACCCGGGGCTGGCCGCCCCGGAAGTGTACCTCTCGCTGGCTCATTACAGGAAAAA
TAAACCTCGGATGGTGGGTTCGCCTAATAGCGGCAACCCCCGGAACGACGCGCCCACTGGTGACGTCGTGCCAGCGATAC
AGGAGTGTGTAGGATCATCTAGGCAAAGCCAGAGTGATGGTTTCTGTTGTGAATTTCCCGGCTGCGGTCGGGTGTTCACA
ACCAAGTCGGGACGTGGGCTCCACCATCGTAAAGCTCACCTAGATTGGTATGACTCCCAGCAGAAGCTTGATTCCGCGAA
AGTGCGCTGGACAAGTGAGGAGTCCGCCCTGATGGCGCGAGAGGAGGCCAAAGTTGCGACGCTCAAACCGCAGCGTATAC
ATCAAGCCCTGTTGTTGGCTCTGCCACACAGGACTCTTGATGCTATTAAAGGCCAAAGGAGGACGGCCCAGTACAAGGAA
CTGGTCCGGCAGGCAGTGGGTGCTCTGTCGGATTCAGGGAAAGGTGCTGGCCCCTCCCAAGCTGAGAGTGTCGGGCTGAA
ACCTCCCTCCCCCCCAGTAGCGCTGGGACCCGGTGACCTTCGGGAATCCGGTGAGCGAGTGCTGGGTGATAAGCCAGGAA
GCTCCCAAGTGTTGGGTGGTGAGGTTCCTACCAAGTCCCCCACCGCCTGCATTAGGGAGCATTTGAGTGCGCTGCCAGAC
AGTCTGCAGCTCGGGCCAGTTGAACGCCGACTGAACCAGGTGATTCGGTCGATAGACGACTGGTCTCGAGAACGCCTGCA
CCAGGAGATAACACGGTATCTCCGCGAGTTCTTTTGGAAACCCCCAACACAAGACCTGGCAAGGGGGCCACTTCCATCTC
AAGACAAGGTGTCTGGGCGCAGACTCAGGCGGGCGGACTATGGACGAGTCCAGAGGGCATGGAAACGGAATCGGAACACA
TGCCTACGTGATCTCATGAGGGACAAGCGCACAGAGTCTGCACCACCCCAAGAACTTATGGTGCCTTATTGGGAGACTGT
GCTACGTTCCGGAGCGTCATGCTCTCCTGGTTTGAGAGAACGGACGGTCGACAAGACTGGGCTCTCTGAGCTATGGGAAC
CGGTGACTTCCAGAGAGGTTGATCGCGCTCTTCCTCCTCTGGGGTCGGCGCCGGGCCCAGACTCCCTGACGCCTGGGGAC
TTCAGGAGGGTATCGTCCAGTGTCTGGGCAAGCATATTTAACATCTTTATGCTGTGTGGGCGGCTCCCGGATTATCTCCT
CGAGTCCCGCACCACACTTATTCCCAAGAAGGACGGTGCTAGCACTCCTGAAGAGTTCAGACCGATTACCGTATCCTCAG
TGGTGGTCAGATGCTTTCACAAGGTGATGGCCAATAGGATGTCCAAAAACATTCCGTTGGACCCTCGCCAGAAAGCCTTT
CGATCTGTGGATGGATGCTCTGAGGGCGTTTTTCTTTTGGATTTCGTTCTCGGGCATGCCAGACGCAACCACCGGCCAGT
GTATCTGGCGTCTTTGGACGTAGCCAAGGCATTTGACCCTGTGTCGCACGCAGCCATCCTTGATGTACTTCGATCATTTG
GGGTTCCTGGCCCCGTGGTCGAGTACATAGCATCTGTGTATGCTGGTTCCAGGACAAGGCTCCAGGGCGCTGGGTGGAAA
TCCCACGCTATTCACCCCACTTGTGGGGTGAAGCAGGGTGATCCGCTCTCGCCCGTGATATTCAACATGGTTATTGACCG
CTTGTTCTCTTTGTTTCCTAAAGAGACGGGAGTTCGAGTGGGAGATGCTGTGTTGAATGGTATGGGGTACGCAGATGACC
TTGTGTTGTTTGCTTCGACACCTGTCGGACTGCAACAGCTGCTGGATATAACTGCCGAATATCTTATGCAGTGTGGTCTG
CGAGTGAATGCTGGGAAATGCTTCACGGTATCCCTTGCGATTGTCCCTCATGAGAGGAAAATGGTGGTCAATTCCCAAGA
ACGATTCAAGTGTCTGGGTCAACCTATTCCTGCTCTAAAGAGGTCTGATCAGTGGAAGTATCTTGGAGTTCCCTTTTCTC
CTGAAGGTCGGCTTAAGGTGGACCCACTTGGCCGCCTGAAGGACGAACTGGAGAAACTTAGAAGGGCTCCATTGAAGCCT
CAGCAGAGGCTTTATGCCTTGCGGACTATGTTAGTTCCGAGCCTCTATCACATGTTGGTATTGGGAGGGACGACCATTAG
TTCGTTGAATAGGCTTGACATTGTGGTTCGGGCCACTGTGCGTAAGTGGCTGGCCCTGCCGCATGATGTACCCAACGCGT
ACATCCACGCTGACGCTCGGGATGGGGGTCTCTCCATTCCCTCCTATCGTTGGAAAGTTCCCCTGCTGCGCTTCCACAGG
CTGAAGGCGCTTTCCCTGCTCTGCAGTGAGACTGGGCCGGAGGATGTGGTTGCGTCTGTGGGGGACGAGATCACACGGGC
AGCCGCTCGTTTGAATGATCATGGTACGACGATCAGTACCAGTAATACCTACAGAGCTCGCTTTGCTAGGTTATTACATA
CTTCCAATGATGGAGCACCTCTTAAAGGGTCGAAGCAGGTGGAGAGCCATCACCGCTGGGTTACCGATGGGACTCTGATG
TTGTCTGGTCGTGATTTTATTGCGTGCAATAGAATCAGGGTCAATGCAATTCCACTGAGGAAGCGATGTGCCCGAGGACG
TGTCCGTGACACTCGTTGCCGTGCGGGATGCGACAACATGGAGACATTACATCACGTGTTGCAGCGATGTCATAGGAACC
ATGATGCAAGAATCAATAAACACGATGCATGTGTGAAGTACCTACTAGATCGGCAACGCTCTCGCGGCAAAAAGGTTTAC
AGGGAACCTCATTTCCGCACGGCAGATGGTCTTTTGAAGCCCGACTCCGTCATCCTTTATGACGCGGCTACCGCGGTAGT
CGTCGATGCTTTGGTTGCCGGGGAAAGGGCTGATCTAGACCGAGAGCATGACCGTAAGGTCCAGAAGTATGCACCACTGA
CCGATCTAGTAAAGGCACTGTACTCTGTCGAAAAGGTGATCTTCTCTTCCATCACAATCAACGCACGGGGGGTGTGGGGA
GGAAAATCATTTAGGAGTCTATCAAAGTTAAGATTGCTTAGTGTCTCTGACGCTAAGGTCCTTTCAACTCGGGTTCTGCT
CGGGGGGGCAGGGGCTGTACGTGTGTTTCATCACCGTATAGCAATCGGTGAGAGAGCTTTTGTCTGATGATTCACACACT
TCTTAGTACAAGTGCTACCTCCTTCCAGCTAGAGGTACAATACAGAGGGTTTTTTAGTGGGTAGTCACCTTGGTGGAGTC
CCACACAACCTGCCACTAGTCACCTGGCAGGAGTGCGTAAAAGCATTTTCCCTATCT
